# Mapping the Architecture of Protein Complexes in *Arabidopsis* Using Cross-Linking Mass Spectrometry

**DOI:** 10.1101/2025.04.28.651104

**Published:** 2025-07-21

**Authors:** Cao Son Trinh, Ruben Shrestha, William C Conner, Andres V Reyes, Sumudu S Karunadasa, Grace Liu, Ken Hu, Shou-Ling Xu

**Affiliations:** 1 Department of Plant Biology, Carnegie Institution for Science, Stanford, California, USA; 2 Carnegie Mass Spectrometry Facility, Carnegie Institution for Science, Stanford, California, USA

**Keywords:** cross-linking mass spectrometry (XL-MS), structural analysis, protein-protein interaction network mapping, gene annotation, Research Reports

## Abstract

Capturing molecular machines in action is essential to understanding the architecture of protein complexes, cellular regulation, and gene function. Here, we present a robust cross-linking mass spectrometry (XL-MS) platform that uses the PhoX cross-linker to map proteome-wide interactions in *Arabidopsis thaliana* under semi-native conditions. Using whole-cell lysates, chloroplasts, and nuclei, we identified 47,119 unique cross-links, including 3,527 inter-protein cross-links. We then constructed a high-confidence protein-protein interaction (PPI) network containing 1,229 proteins and 1,446 PPIs. In silico validation using STRING confirmed 637 heteromeric interactions with a combined confidence score of at least 0.4, including 532 interactions with a score of at least 0.7. The remaining interactions are novel. Our dataset provides direct experimental evidence of physical interactions and defines residue-level binding interfaces. XL-MS resolved the spatial topology of structurally challenging protein complexes, such as the chloroplast 70S ribosome stalks, and transient intermediates involved in photosystem repair. XL-MS also revealed both conserved and distinct interaction patterns across photosystem complexes, ribosomes, and chromatin-associated proteins. Notably, the data suggest that the ribosome undergoes ubiquitination and is regulated by the 26S proteasome. This finding is further supported by *in vivo* diGly proteomic analysis. Among the novel interactions uncovered is a metabolite enzyme that interacts with histones, which could potentially contribute to histone modification. This work establishes XL-MS as a powerful tool for advancing the functional annotation of genomes and the understanding of the architecture of protein complexes and cellular regulation in plants, providing a valuable resource for the plant research community.

## Introduction

Understanding how molecular machines operate within cells - and capturing them in action - is essential to unraveling the principles of life and gene function. A powerful tool for this purpose is cross-linking mass spectrometry (XL-MS), an emerging high-throughput approach that enables the capture of protein complexes in their native context across diverse organisms (1, 2). Unlike affinity purification mass spectrometry (AP-MS) and proximity labeling mass spectrometry (PL-MS) (3–5), XL-MS does not require antibodies or genetically tagged transgenes. Instead, it relies on chemical cross-linkers that covalently link spatially proximal residues within or between proteins in solution, followed by protease digestion and mass spectrometric identification of the resulting cross-linked peptides. In addition to generating a “parts list” of interacting proteins, XL-MS provides direct evidence of physical interactions and precisely maps contact sites at the amino acid level, providing unparalleled structural insight into macromolecular assemblies.

Despite its promise, large-scale proteome-wide XL-MS - such as in whole-cell lysates, isolated organelles, or *in vivo* systems - remains challenging due to the inherent low abundance and heterogeneity of cross-linked peptides and the complexity of data analysis (1, 6). In plants, additional barriers further complicate its application, including rigid cell walls and large central vacuoles that dilute cross-linkers, as well as secondary metabolites that can chemically interfere with cross-linking chemistry (7, 8). Recent pioneering studies in Arabidopsis, algae, and soybean have demonstrated the potential of XL-MS in plant systems (7, 9, 10), but its overall implementation remains limited.

To address these challenges, we developed a cross-linking strategy optimized for proteome-wide mapping in complex Arabidopsis samples using the enrichable cross-linker PhoX. PhoX is a phosphonic acid-based reagent that, like BS3 and DSSO, contains NHS esters and primarily targets lysine residues (11). Its phosphonic acid handle allows efficient enrichment by immobilized metal affinity chromatography (IMAC). With a maximum range of 35Å - consisting of a 20 Å Cα-Cα distance from its rigid structure and an additional 15 Å to account for protein flexibility - PhoX is well suited for high confidence, large-scale structural proteomics (12). To reduce sample complexity and improve cross-linking efficiency, we used subcellular fractionations. To further improve the detection of the low abundance cross-linked peptides, we performed extensive high-pH reversed-phase HPLC fractionation after IMAC enrichment. Together, these measures allowed for comprehensive proteome-wide XL-MS mapping.

We identified a total of 47,119 unique cross-linked peptide pairs from cross-linked cell lysates, chloroplasts, and nuclei, including 43,592 intra-protein (self-link) and 3,527 inter-protein cross-links. These results enabled us to construct a PPI network comprising 1,229 proteins and 1,446 interactions, of which 1,390 are heteromeric and 56 are homomultimeric. STRING analysis, which catalogs only heteromeric PPIs, validated 637 interactions with a combined confidence score of at least 0.4. The rest represent previously uncharacterized interactions. Our dataset provides experimental evidence of direct physical binding and defines protein binding interfaces with amino acid resolution for both known and novel interactions.

Beyond interaction mapping, our XL-MS data provide structural and functional insights into key cellular processes. By resolving flexible or transient components within the 70S chloroplast ribosome and photosystem complexes, our results complement existing cryo-EM structures. XL-MS also enabled a comprehensive mapping of the photosystem, 80S ribosome, and histone interactomes within heterogeneous protein populations, revealing structural biology and capturing both known and novel interactions. We identified a metabolic O-acyltransferase that interacts with histones, suggesting a potential new mechanism of chromatin regulation via post-translational modification. Together, these results improve protein annotation, shed light on protein complex architectures, and provide as a valuable resource for the broader research community - laying the foundation for future discoveries.

## Results

### Efficient cross-linking workflow for complex protein samples identifies numerous cross-links in Arabidopsis

**Figure 1.**
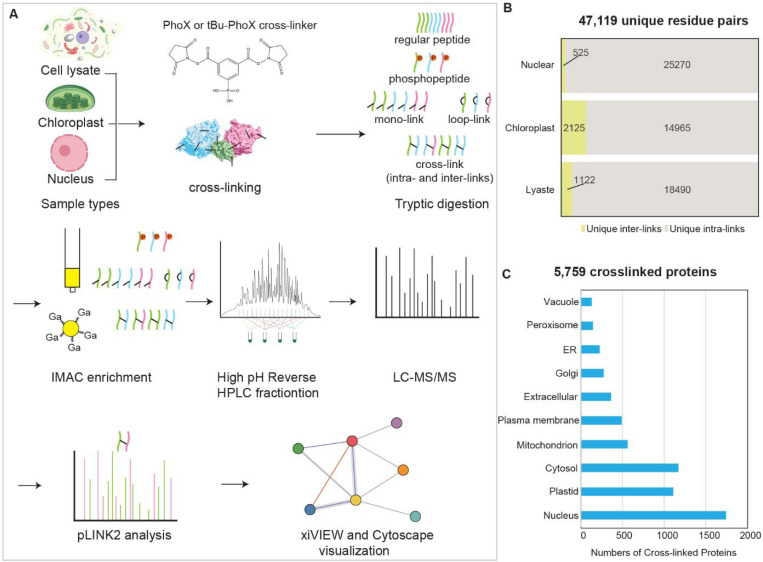
Efficient cross-linking workflow for complex protein samples identifies numerous cross-links in Arabidopsis. (**A**) Schematic of the workflow using PhoX or tBu-PhoX to covalently cross-link spatially proximal lysine residues in Arabidopsis subcellular fractions (lysate, chloroplast, nucleus), followed by digestion, IMAC enrichment, high pH reversed-phase HPLC fractionation, and LC-MS/MS. Data were analyzed using pLINK2, Cytoscape, and xiVIEW. (**B**) Summary of unique intra- and inter-protein cross-links from each subcellular fraction. Intra-links connect non-overlapping peptides within a protein; inter-links include heteromeric and homomultimeric cross-links. (**C**) Subcellular localization of cross-linked proteins based on SUBA5, with redundancy of proteins assigned to multiple compartments.

To gain structural insights and visualize molecular machines in their native cellular context within Arabidopsis cells, we performed XL-MS (see Methods) on fractionated seedling samples, including cell lysates and chloroplasts from light-grown seedlings and nuclei from dark-grown seedlings. Using the PhoX cross-linker (Fig. 1A), we established a robust pipeline optimized for proteome-wide detection of low-abundance cross-linked peptides in complex Arabidopsis samples.

Optimization of the PhoX concentration showed a shift to higher molecular weights at 2–5 mM (red arrows, Fig. S1A-D), indicating successful cross-linking, with no additional improvement at higher concentrations. Based on these results, we used 2 and 5 mM PhoX for subsequent experiments (see Methods). After cross-linking, we precipitated the proteins with phenol, digested them with trypsin, and enriched the cross-linked peptides using GaCl_₃_-based IMAC (13). We then fractionated the enriched peptides by high-pH reversed-phase chromatography, analyzed them by LC-MS/MS, and identified cross-links using pLINK2 (14). High-resolution, high-accuracy data for both MS1 (precursor ions) and MS2 (fragment ions) were acquired on an Orbitrap Eclipse mass spectrometer to ensure reliable cross-link identification.

To optimize data acquisition, we compared the standard XL workflow with real-time spectral library search (RTLS) (15), both targeting highly charged precursors (+3 and above). RTLS uses a survey MS2 scan to reduce mono-link detection. In cell lysates, RTLS reduced mono- and loop-link identifications by ~50% and ~31%, respectively (Fig. S2), but also significantly reduced intra-link (43%) and inter-link (40%) identifications. Because of this trade-off, we used the standard XL workflow to analyze cross-linked chloroplast and nuclear samples.

In total, we identified 47,119 unique cross-linked peptide pairs (intra- and inter-links) representing 5,759 proteins. Specifically, we detected 25,795, 17,090, and 19,612 cross-links from the nucleus, chloroplast, and cell lysate, respectively (Fig. 1B and. S3, Table S1). Subcellular localization analysis using SUBA5 revealed that the cross-linked proteins were distributed in various cellular compartments, including the nucleus, plastid, cytosol, mitochondria and other organelles (Fig. 1C, Table S2).

To assess the quality of cross-linked peptide identification, we examined the identified cross-links (Table S1) and found consistent peptide pairs mapping to the same sites or regions. For example, in ribosomal subunits, we detected site-specific cross-links with variable miscleavages, such as those between uL15x-K92 and eL13z-K166/K172 (Fig. S4A). Conserved region cross-links were also observed, such as VHA-E3-K49 with both VHA-B1-K437 and VHA-B2-K438 (Fig. S4B), and TOP1A-K672 with ERD14-K38 and COR47-K46 (Fig. S4C). These independent and consistent observations underscore the robustness and reliability of our results.

### Validation of intra- and inter-protein cross-links by structural mapping

**Figure 2.**
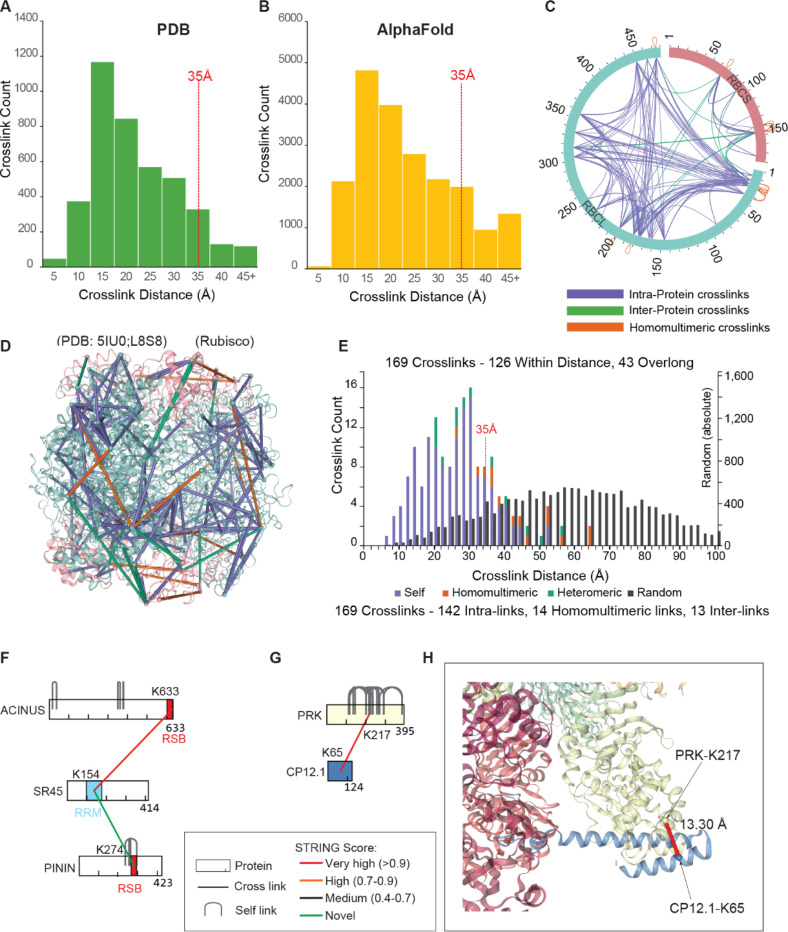
Validation of intra- and inter-protein cross-links by structural mapping. (**A**-**B**) Histogram of Euclidean Cα-Cα distances of intra-protein cross-links mapped to Arabidopsis structures from PDB (A) or AlphaFold (B), showing 94% and 89% within the 35Å constraint, respectively. (**C**-**E**) Cross-links mapped to the Arabidopsis Rubisco L_8_S_8_ complex modeled from the L_2_S_2_ structure (PDB:5IU0). RBCL is shown in cyan and RBCS is shown in pink. Self(intra), heteromeric, and homomultimeric links are shown in purple, green, and orange, respectively. The circular view (C) and structural mapping (D-E) show that 74.56% of the cross-links are within 35Å. (**F**) Cross-links between SR45 and ACINUS or PININ occur at known interaction interfaces involving the RRM domain of SR45 and the RSB domain of ACINUS and PININ. (**G-H**) Intra- and inter-protein cross-links between PRK and CP12.1 mapped onto the GAPDH-CP12-PRK complex (PDB:6KEZ), aligning with the known interaction interface.

To evaluate the validity of the cross-links, we first used ComMap (16) to map intra-links to high-resolution protein structures available in the Protein Data Bank (PDB) and predicted structures by AlphaFold. Specifically, 721 Arabidopsis proteins had high-resolution structures in the PDB, and 3,975 were available through AlphaFold (Table S1). A total of 21,981 cross-links were successfully mapped, with 94% of the PDB-mapped cross-links and 89% of the AlphaFold- mapped cross-links satisfying the 35 Å distance constraint (Fig. 2A-B).

To evaluate both intra- and inter-protein cross-links, we mapped 174 cross-links onto the L8S8 Rubisco assembly modeled from the Arabidopsis L2S2 crystal structure (PDB:5IU0) (17). Of these, 169 cross-links were mapped to the large (479 aa) and small (181 aa) subunits, while five could not be mapped because the PDB structure lacks the N-terminal region (residues 1–11) (Fig. 2C). The majority (126, 74.6%) of mapped cross-links fell within the expected Cα-Cα distance of ≤35 Å; the remaining 43 (25.4%) exceeded this range, suggesting regions of structural flexibility (Fig. 2D–E). Similar mapping results were obtained using the hornwort Rubisco cryo-EM structure (Fig. S5A-B), further supporting the validity of our cross-linking data.

To assess whether cross-linked lysine residues are positioned within predicted interaction interfaces, we examined several PPIs using available structural data. The cross-link between ACINUS-K633 and SR45-K154 maps near the RSB domain of ACINUS (aa 599–631) and within the RRM domain of SR45 in the ASAP core complex (PDB:4A8X) (18–20) (Fig. 2F, Fig. S6A). Similarly, the cross-link between SR45-K154 and PININ-K274 aligns with the same RRM domain and the predicted RSB domain of PININ (Fig. 2F, Fig. S6B). For PRK-K217 and CP12.1-K65, the cross-link localizes to the interaction interface in the GAPDH-CP12-PRK complex (PDB:6KEZ) (21) with a Cα-Cα distance of 13.3 Å (Fig. 2G-H). These results confirm that the cross-linked residues are located at or within a few amino acids of the interaction interfaces, consistent with previous observations (22).

### XL-MS systematically identifies direct protein-protein interactions and reveals known and novel complexes

**Figure 3.**
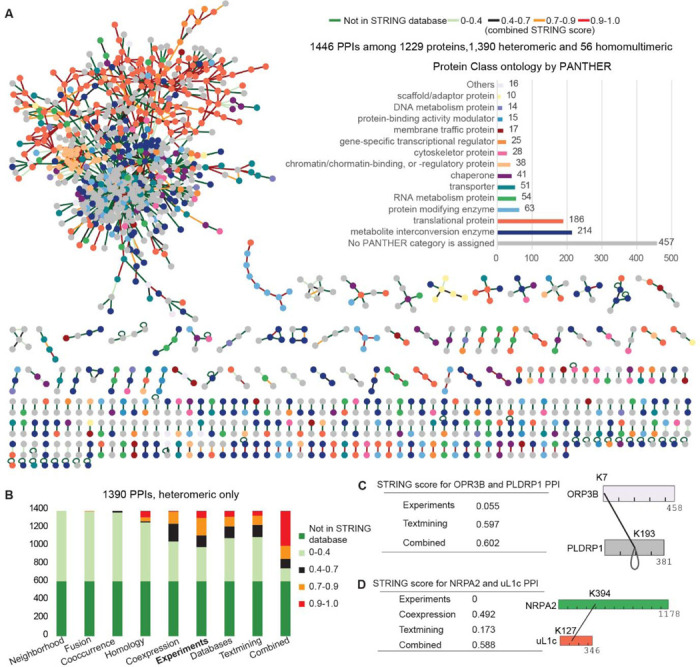
XL-MS identifies direct protein-protein interactions and reveals known and novel complexes. (**A**) XL-MS identified an Arabidopsis interactome comprising 1,446 PPIs, including 1,390 heteromeric and 56 homomultimeric interactions among 1,229 proteins. Nodes represent proteins, colored by PANTHER protein class; edges represent interactions, colored by the combined STRING confidence score. Of the total PPIs, 637 PPIs have a combined confidence score of at least 0.4, including 532 with a score of at least 0.7. (**B**) Summary of the STRING score, showing the contribution from eight evidence sources. XL-MS provides experimental support for 71% of the heteromeric PPIs that either have low confidence scores (<0.4) or lack prior experimental evidence. (**C-D**) Examples of specific PPIs detected by XL-MS that complement low confidence or missing experimental STRING scores: the ORP3B-PLDRP1 interaction (cross-linked at ORP3B-K7/PLDRP1-K193) and the NRPA2-uL1c interaction (NRPA2-K394/uL1c-K127).

We imported 3,527 inter-protein cross-links into Cytoscape (23), revealing 1,446 PPIs among 1,229 proteins, including 1,390 heteromeric and 56 homomultimeric interactions (Fig. 3A, Table S1). Proteins were classified using the PANTHER protein class tool, and nodes were colored accordingly (24). Many interacting proteins shared the same classification, supporting the reliability of our PPI map. Notably, 457 of the 1,210 proteins involved in heteromeric interactions lacked PANTHER annotations, contributing to 819 interactions - 369 between unclassified proteins and 450 between classified and unclassified proteins.

We also incorporated STRING confidence scores (25) into the network and color-coded the edges accordingly (Fig. 3A). These scores combine evidence from eight sources (Fig.3B) (25). For heteromeric PPIs, 38.2% of PPIs had high STRING confidence: 388 scored ≥ 0.9 and 144 scored 0.7–0.9. The remainder included 105 medium (0.4–0.7), 144 low (<0.4), and 609 with no STRING score (Fig.3B). The PANTHER and STRING results indicate that both known and novel PPIs were captured, highlighting the power of systematic XL-MS to reveal previously uncharacterized protein complexes and pathways.

Even among PPIs with good STRING combined scores, many lack experimental support for direct or indirect interactions in Arabidopsis or homologs in other species. Among the heteromeric PPIs identified, only 28.9% (n = 402) have experimental support (score ≥ 0.4), while 71.1% have low (<0.4) or no scores (Fig. 3B). Our XL-MS data provide evidence for their direct interactions and contact sites. For example, ORP3B (AT3G09300, oxysterol-binding protein) and PLDRP1 (AT5G39570, PLD-regulated protein 1) have a combined score of 0.602 but a low experimental score (0.055); we identified a cross-link between ORP3B-K7 and PLDRP1-K193 (Fig. 3C), suggesting a common role in lipid trafficking and signaling. Similarly, NRPA2 (AT1G29940, RNA polymerase predicted to localize to both the nucleus and chloroplasts) and uL1c (AT3G63490, chloroplast 50S ribosomal protein) have a combined score of 0.588 but no experimental support; a cross-link between NRPA2-K394 and uL1c-K127 suggests a direct interaction and potential coupling of chloroplast transcription and translation (Fig. 3D).

### XL-MS resolves the topology of chloroplast 70S ribosome stalk regions inaccessible by Cryo-EM

**Figure 4.**
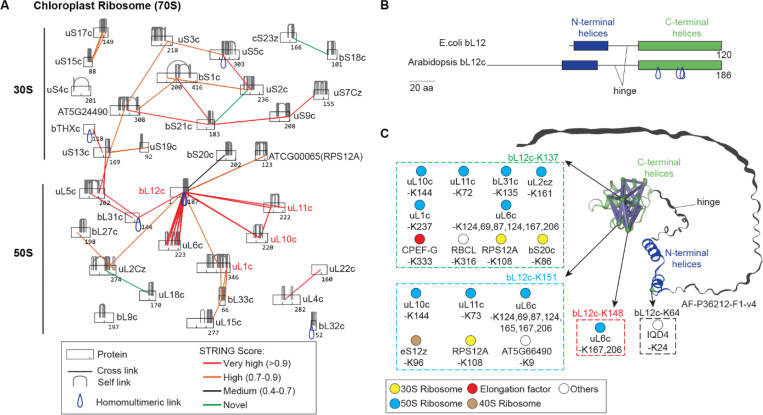
XL-MS resolves the topology of chloroplast 70S ribosome stalk regions inaccessible by cryo-EM. (**A**) XL-MS identifies flexible stalk proteins (bL12c, uL1c, uL10c, uL11c), with cross-links color-coded by STRING confidence. (**B**) Domain comparison between Arabidopsis bL12c and *E. coli* bL12. Homodimer interfaces are found at the C-terminus of bL12c. (**C**) Cross-links mapped onto the predicted bL12c structure (AF-P36212-F1-v4) showing lysines K64, K137, K148, and K151 interacting with ribosomal subunits and the elongation factor CPEF-G. Nodes are color-coded according to annotation category.

We mapped the topologies of several protein complexes (Fig. S7A-G), including the flexible stalk regions of the chloroplast 70S ribosome (Fig. 4A-C), which have remained difficult to resolve due to the dynamic nature of proteins such as uL1c, uL10c, uL11c, and bL12c (26, 27). In total, we identified 424 cross-links across 34 ribosomal subunits of the 70S ribosome, including 352 intraprotein, 64 heteromeric, and 8 homomultimeric cross-links. Notably, many intra- and interprotein cross-links involved these flexible stalk proteins. When cross-links were mapped to spinach 70S ribosome structures (PDB: 5MMM and 5X8P; Fig. S8A-B), most cross-links involving uL1c, uL10c, uL11c, and bL12c could not be precisely assigned to specific locations within the stalk regions because these proteins are either missing or only partially resolved in the cryo-EM structures.

Arabidopsis bL12c has a plant-specific disordered N-terminus, followed by conserved structured N-terminal (NTD) and C-terminal (CTD) domains connected by a flexible hinge (Fig. 4B). Essential for translation, bL12c uses hinge-mediated mobility to recruit specific translation factors(28). XL-MS identified interactions between bL12c (the two highly homologous proteins AT3G27850/bL12cx and AT3G27830/bL12cz) and several ribosomal core proteins, as well as the elongation factor CPEF-G, mainly through its structured CTD (Fig. 4C). Although bL12c dimers are typically mediated by the NTD (29), XL-MS revealed unexpected homodimer cross-links within the CTD - the same region involved in translation factor binding (Fig. 4C). This suggests that CTD dimerization may serve as an interface for the recruitment of interaction partners. Taken together, these results highlight both conserved and plant-specific features of bL12c and demonstrate the power of XL-MS in resolving dynamic aspects of ribosomal architecture.

### XL-MS reveals dynamic connectivity and unique features in photosystem complexes

Photosystems I and II (PSI and PSII) and their associated light-harvesting complexes (LHCI and LHCII) are essential for light energy harvesting and carbon fixation, but they require continuous repair, especially under high and fluctuating light conditions (30–32). Although progress has been made in understanding certain steps of the PSII repair (33, 34), many aspects, including the detailed mechanism, identification of repair intermediates, and specific contributing factors, remain unresolved.

**Figure 5.**
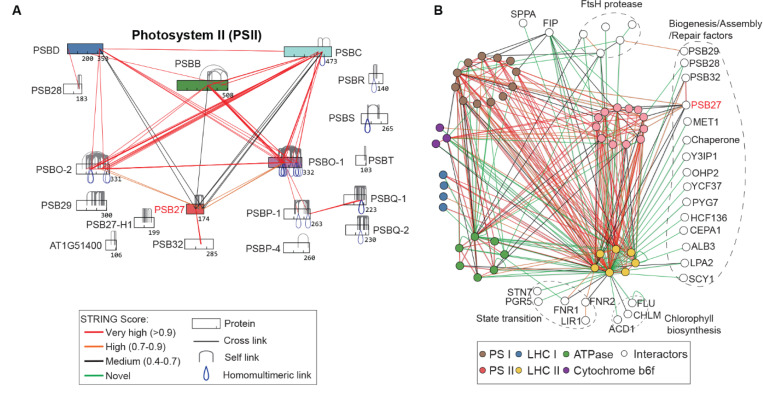
XL-MS reveals dynamic connectivity and unique features in photosystem complexes. (**A**) PSII topology showing cross-links color-coded by STRING confidence. Notable PSB27 cross-links (K93, K168) highlight interactions with PSII core and OEC components. (**B**) XL-MS uncovers extensive interactions among PSI, PSII, LHCI, LHCII, ATPase, FtsH/SPPA proteases, and transient proteins involved in state transitions, assembly, repair, and chlorophyll metabolism.

Using XL-MS, we identified extensive interactions involving the PSII repair factor Psb27 (AT1G03600) (Fig. 5A). Unlike its cyanobacterial counterpart, which functions in both assembly and repair, Arabidopsis Psb27 appears to be specialized for repair. In contrast, the less homologous Psb27-H1(AT1G05385) is involved in assembly. Notably, Arabidopsis Psb27 lacks a lipid anchor and is structurally more flexible than the cyanobacterial version (35) (Fig. S9A). A partial structure from Chlamydomonas (PDB: 6KAC) captured residues 91–114 of Psb27 (36). Our XL-MS data revealed that Psb27 interacts with both the PSII reaction center and the oxygen-evolving complex (OEC), involving K93 (as in 6KAC) and a second site at K168 (Fig. 5A, Fig. S9B, S10A-H). These distance constraints (Fig. S9B) suggest that Psb27 engages PSII in a more dynamic and flexible manner in plants.

XL-MS also revealed extensive interactions between PSI, PSII, LHCI, LHCII, ATP synthase, ATP-dependent proteases (FtsH, SPPA) and transient proteins involved in state transitions, complex assembly and repair (Fig. 5B). Notably, chlorophyll biosynthesis and catabolic proteins (e.g., ACCELERATED CELL DEATH 1 (ACD1), magnesium protoporphyrin IX O-methyltransferase (CHLM), FLUORESCENT IN BLUE LIGHT (FLU)) were found to directly associate with LHCII, suggesting coordinated chlorophyll delivery and reactive oxygen species (ROS) control during LHCII assembly. In addition, many assembly and repair factors were found to associate with LHCII, indicating their readiness for rapid engagement during dynamic photosystem remodeling.

### XL-MS of ribosome complexes reveals conserved ribosome-associated factors

**Figure 6.**
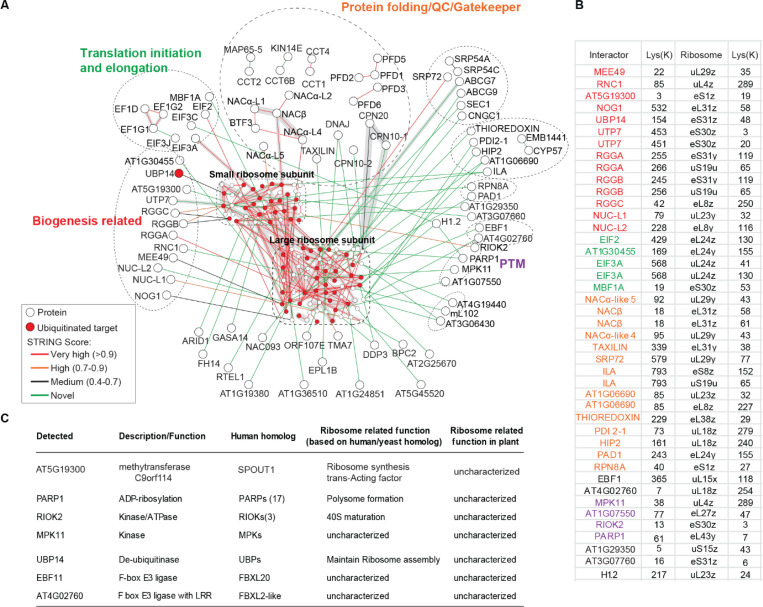
XL-MS of ribosome complexes reveals conserved ribosome-associated factors. (**A**) XL-MS identified extensive interactions within ribosomal subunits as well as with proteins involved in ribosome biogenesis, translation, folding, quality control, stress response, and other regulatory pathways. Ribosomal proteins are overlaid with ubiquitination data; red nodes indicate ubiquitinated proteins. (**B**) Table showing covalently cross-linked lysine residues between ribosomal proteins, reflecting spatial proximity. Protein labels are color-coded according to the functional categories shown in (A). (**C**) Table of XL-MS-identified ribosome-associated interactors with potential post-transcriptional or post-translational regulatory functions, many of which remain uncharacterized in plants.

We identified extensive interactions involving the 80S ribosome, a complex of 60S and 40S subunits that synthesizes nuclear-encoded proteins. Ribosomes assemble in the nucleolus, localize to the cytoplasm, and associate with the endoplasmic reticulum (ER). As a dynamic macromolecular machine, the ribosome coordinates translation with numerous proteins and RNAs. Errors in translation that produce misfolded proteins, or defects in the ribosome itself, can trigger ribosome-associated quality control pathways (37). Using XL-MS, we mapped detailed structural information within this highly heterogeneous complex, capturing not only intra- and inter-subunit contacts (Fig. S11A-D), but also interactions with proteins involved in ribosome biogenesis, translation, folding, quality control, stress response, and other regulatory pathways (Fig. 6A). These interactions were resolved at the amino acid level, providing detailed insight into the architecture of the functional interface (Fig. 6B, Table S4).

While many interactions showed good STRING scores (≥0.4), our analysis also revealed numerous novel interactions (green edges) between specific protein pairs. Although homologs of these proteins have been studied in other organisms for their ribosome-related functions, their specific roles in plants remain largely uncharacterized. Our XL-MS data suggest conserved functions: for example, the UTP7 homologs AT3G10530 and NOG1–1 may contribute to ribosome biogenesis (38, 39), whereas AT5G50840 (α-taxilin-like) and ILITHYIA(ILA) may be involved in protein folding or translation (40, 41). MBF1A, a transcriptional coactivator, may be involved in stress responses at stalled ribosomes, similar to its role in yeast (42) . Other candidates include a methyltransferase potentially involved in rRNA processing and polysome assembly (43, 44) , a RIO kinase associated with ribosome biogenesis (45, 46), and PARP1, which may regulate translation via ADP-ribosylation (47) (Fig. 6C). By mapping these interactions to specific ribosomal proteins, our XL-MS dataset provides a valuable resource for future functional studies of ribosome-associated factors in plants.

We identified several interactors related to ubiquitination, including two E3 ligases (EBF1 and AT4G02760) and one deubiquitinase UBP14. In addition, we detected direct interactions between ribosomes and 26S proteasome components, such as RPN8A and PAD1 (Fig.6A). Given the need for active ribosome turnover to maintain quality control and these XL interaction data, we hypothesized that ribosome proteins undergo *in vivo* ubiquitination as a regulatory mechanism. To test this hypothesis, we conducted a diGly ubiquitin proteomic analysis (48), which revealed the *in vivo* ubiquitination of 37 large and 27 small ribosomal subunits (Fig. 6A, Table S4). These findings suggest that the ribosomes are ubiquitinated *in vivo* and may be targeted for degradation by the 26S proteosome.

### Histone interactome reveals conserved and novel chromatin-associated interactions

**Figure 7.**
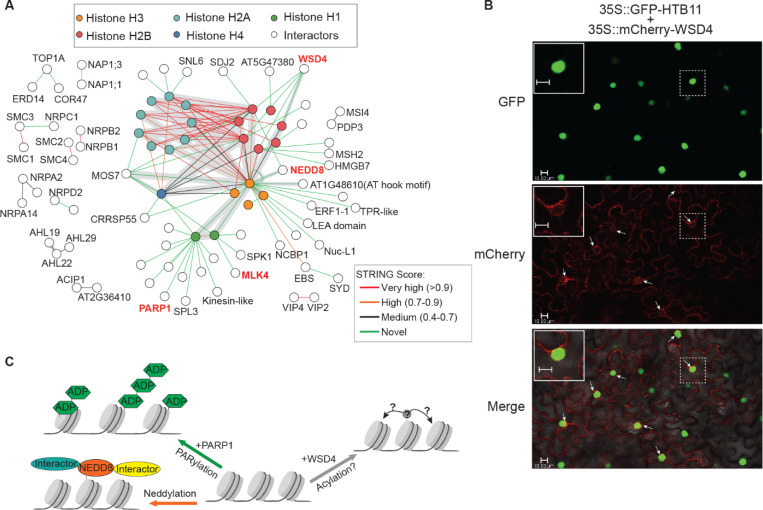
Histone interactome reveals conserved and novel chromatin-associated interactions. (**A**) XL-MS reveals extensive interactions between histones and both known and previously uncharacterized proteins, including components of chromatin-associated complexes. Enzymatic regulators are highlighted in red. (**B**) Visualization of the co-localization of GFP-HTB11 and mCherry-WSD4 in *N.benthamiana.* Green: GFP-HTB11; red: mCherry-WSD4. Overlapping signals are indicated by arrows, with a zoom-in view highlighted in the inset. Scale bar=10μm. (**C**) XL-MS identifies interaction between histones and PARP1, NEDD8 and WSD4, suggesting their potential roles in histone modifications and polynucleosome organization. Multiple cross-links between histone H2B proteins and WSD4, an O-acyltransferase, support a possible function for WSD4 in histone acylation.

Our XL-MS analysis revealed extensive interactions between histones and both known and novel interactors, providing new insights into the networks involved in gene expression and chromatin regulation (Fig. 7A). We identified several nuclear complexes associated with chromatin, including nucleosome assembly proteins (NAPs), chromosome structure maintenance proteins (SMCs), nuclear RNA polymerase components (NRPs), chromatin regulators (PWWP DOMAIN PROTEIN 3(PDP3), MULTICOPY SUPPRESSOR OF IRA1 4(MSI4)), transcriptional activator and acetylation reader (AT2G36410 and ACETYLATED INTERACTING PROTEIN 1(ACIP1)), and subunits of the PAF complex (VERNALIZATION INDEPENDENCE 2 and 4 (VIP2, VIP4)) (Fig. 7A).

Although many proteins are known to influence chromatin architecture and gene regulation in plants, their direct interactions with histones and the nature of their contact sites remain largely uncharacterized. Using XL-MS, we identified a diverse array of proteins that interact with histone, including chaperones such as SUVH1/3-interacting DNAJ domain-containing protein 2(SDJ2) and NUCLEOLIN 1(NUC-L1)), chromatin remodeling factors such as EARLY BOLTING IN SHORT DAYS(EBS), SPLAYED(SYD) and SPIKE1(SPK1)), transcription factors such as SQUAMOSA PROMOTER BINDING PROTEIN-LIKE 3(SPL3), co-regulators such as SIN3-LIKE 6(SNL6), DNA repair proteins such as MUTS HOMOLOG 2 (MSH2), and structural proteins such as HIGH-MOBILITY GROUP BOX 7 (HMGB7). Notably, we found that MOS7, a nucleoporin homologous to the human NUP88, cross-linked with several histones. This provides direct evidence of nuclear envelope-histone interactions that may contribute to chromatin organization in plants (49). In addition, we identified histone interactions with several enzymatic regulators, including the kinase MLK4, the poly(ADP-ribose) polymerase PARP1 (both of which were cross-linked to H1), and the ubiquitin-like protein NEDD8 (which was cross-linked to H2B and H3).

Surprisingly, XL-MS identified four distinct cross-links between histone H2B and the O-acyltransferase WSD4 (Fig. 7A and S12A-E). Members of the WSD family, including WSD4, are traditionally associated with lipid metabolism and function as wax ester synthase/acyl-CoA-dependent O-acetyltransferases (50, 51). However, WSD4 is preferentially expressed in roots, which is unusual because wax deposition is uncommon in these tissues (51). This suggests that WSD4 may have an alternative function unrelated to wax biosynthesis. Notably, TAIR predicts that WSD4 is localized in nucleus. To test whether WSD4 co-localizes with histones, we co-expressed mCherry-WSD4 and GFP-HTB11 in *Nicotiana benthamiana*, and observed overlapping nuclear signals, as indicated by the arrows in Fig. 7B. Together, the XL-MS and co-localization data raise the intriguing possibility that WSD4 may catalyze histone acylation, a hypothesis that warrants further investigation (Fig.7C).

Taken together, these findings reveal previously uncharacterized histone-associated proteins and enzymatic activities in plants, providing new insights into chromatin biology and laying the groundwork for future studies of plant gene regulation.

## Discussion

Systematic mapping of PPIs has greatly advanced our understanding of cellular function, but plant PPI networks remain largely unexplored (5, 52). While comprehensive maps exist for humans and other model organisms (53–55), similar efforts are just beginning in plants (56–58). This gap represents a critical opportunity, as plants are central to addressing global challenges such as food security, climate change, and sustainability. Expanding proteome-wide PPI mapping and structural biology studies in plants will accelerate our mechanistic understanding of cellular processes and drive advances in agriculture and biotechnology.

We developed a systems biology approach to capture molecular machines in action by mapping PPIs and their interfaces using XL-MS. To address the inherent challenges of XL-MS in complex biological samples, we established an optimized pipeline that integrates the PhoX crosslinker, subcellular fractionation, and high-pH reversed-phase HPLC offline fractionation. This workflow enabled the identification of over 47,000 crosslinks. The high quality of the data was confirmed by mapping the intralinks onto experimentally determined structures in the PDB and predicted models from AlphaFold.

Our XL-MS analysis revealed the topological organization of multiple protein complexes, including the 70S ribosome, 26S proteasome, photosystems I and II, ATP synthases, and chromatin-associated factors. We demonstrate that XL-MS is highly effective in resolving spatial relationships within dynamic and heterogenous assemblies. Notably, we mapped flexible regions of the 70S ribosome stalk and established connectivity for components that are missing or only partially resolved in the existing cryo-EM structures. This includes flexible elements such as BL12c, for which we discovered unexpected C-terminal domain (CTD) dimerization within the translation factor binding region – suggesting that CTD dimerization may serve as an interface for recruiting interaction partners. Additionally, we mapped interactions involving transient repair proteins in PSII. Together, these results highlight the unique strength of XL-MS in complementing cryo-EM by providing structural insights into conformationally dynamic regions, underscoring the value of our approach in advancing in situ structural biology.

Our XL-MS analysis identified 1,446 high-confidence PPIs among 1,229 proteins, establishing a comprehensive interaction map in *Arabidopsis*. Functional categorization using PANTHER and network analysis via STRING analysis revealed that these interactions span a wide range of biological processes. Notably, 637 heteromeric PPIs had a combined STRING confidence score ≥ 0.4, including 532 with score ≥ 0.7, underscoring the robustness of our interaction mapping. In addition, we identified 56 homomultimeric PPIs, which are not represented in STRING, as the database catalogs only heteromeric interactions. Recent studies suggest that dimerization is essential for the function of many proteins (59, 60).

Further validation of our dataset is provided by genetic evidence: mutations in several newly identified interacting pairs result in shared or opposing phenotypic outcomes. For example, the chromatin remodeling protein EBS promotes early flowering, whereas its interactor SYD, a component of the chromatin structure remodeling complex, delays flowering when mutated(61, 62). In another example, loss-of-function mutations in both FLK (a flowering locus KH domain protein) and its interactor KHZ1 (a CCCH zinc finger and KH domain protein) result in late flowering (63, 64). These phenotype correlations support the functional relevance of the identified interactions and highlight the potential of XL-MS-based interaction maps to accelerate gene discovery.

In addition, the interaction between ribosomal proteins and E3 ligases, deubiquitinases, and 26S proteasome components, such as RPN8A and PAD1, suggests that ribosomes may be regulated by ubiquitin-mediated pathways. Our ubiquitin diGly proteomics analysis supports this hypothesis by identifying the *in vivo* ubiquitination of 37 large and 27 small ribosomal subunits. These identifications were of high confidence, with many di-Gly peptides detected in both light (^14^N) and heavy (^15^N) forms (65, 66). Together with the XL-MS data, these findings imply the existence of a potential ribosome quality control mechanism similar to those pathways described in yeast (67). The exact role of E3 ligases and deubiquitinases remains to be confirmed functionally. EBF1 has been shown to regulate ethylene signaling by targeting EIN3/EIL1 for degradation (47); however, its ribosome-related functions have yet to be explored. UBP14 is essential for development in Arabidopsis but dispensable in yeast (48).

XL-MS uncovered a wealth of novel PPIs, with over half not previously annotated in the STRING database. For example, we identified both well-established and unreported PPIs involving ribosomal proteins and histones (see Figs. 6A and 7A), expanding our understanding of these conserved molecular machines across species (68, 69). We also uncovered a broad array of plant-specific PPIs, particularly within chloroplasts (Fig. 5B), reflecting unique features of the plant cellular machinery.

Among the histone interactors, we identified several enzymatic regulators, including MLK4 and PARP1 (cross-linked to H1), and NEDD8 (cross-linked to H2B and H3). Although PARP1 and NEDD8 are known histone modifiers in other systems, their roles in plants remain undefined (70–73). Previous studies have shown that MLK4 phosphorylates H2A and regulate flowering (74); thus, it may have broader functions in histone regulation.

We identified WSD4, an O-acyltransferase, as a potential novel histone regulator. Four independent cross-links were identified between WSD4 and H2B, and their interaction was further supported by nuclear co-localization. Although O-acyltransferase are usually linked with lipid metabolism(50), their function in protein modification remains largely unknown. Given the known examples of protein acylation by metabolic enzymes and the regulatory role of lysine acylation(75–78), we propose that WSD4 may catalyze histone acylation. This would reveal a previously unrecognized chromatin regulatory mechanism.

Despite decades of study, many *Arabidopsis* genes remain uncharacterized (79). Our work demonstrates that XL-MS efficiently maps PPIs under semi-native conditions, accelerating gene discovery and functional annotation. In addition, the extensive intra-protein cross-links generated in this study can aid in developing structural prediction tools such as AlphaFold. This dataset is a valuable resource for Arabidopsis functional genomics, and this approach can be applied to other species, particularly crops. Further developing this technique will advance comparative proteomics and deepen our understanding of fundamental biological processes.

## Material and methods

### Plant growth condition

Arabidopsis thaliana wild-type (Col-0) seeds were sterilized and sown on ½ Murashige and Skoog (MS) agar square plates supplemented with 0.6% (w/v) phytoblend and lined with nylon fabric. After stratification at 4°C for 3 days, the plates were transferred to growth conditions.

Light-grown seedlings: plants were vertically grown in a growth chamber at 22°C under a 16-hour light / 8-hour dark cycle for 10–12 days, then flash frozen in liquid nitrogen and stored at −80°C for crosslinking experiments on cell lysates and isolated chloroplasts. Dark-grown seedlings: plants were grown horizontally at 22°C in complete darkness for 3 days in a growth chamber, then flash frozen in liquid nitrogen and stored at −80°C for nucleus isolation. Dark growth was used to reduce Rubisco content.

Frozen Arabidopsis tissue was cryo-ground to a fine powder. Different amounts of tissue were used for cross-linking experiments: 6 g from 10 d seedlings for cell lysates, 195 g from 12d seedlings for chloroplasts, and 32 g (replicate 1 with PhoX) or 14 g (replicate 2 with tbu-PhoX) from 3d dark-grown seedlings for nuclei.

### Fraction of plant seedling tissues for cell lysate, chloroplast and nucleus

#### Cell lysates:

Homogenized 10 d old light growth tissues were extracted using MOPS buffer [100 mM MOPS pH 7.6, 150 mM NaCl, 1% Nonidet P-40, 1 mM PMSF, 1x Roche protease inhibitor] at a tissue:buffer ratio of 1:1 (g/mL, w/v). The samples were centrifuged at 20,000 g for 15 min at 4^o^C, and the supernatant was then filtered twice with Miracloth and used for the cross-linking reaction.

#### Chloroplast isolation:

Frozen, homogenized tissue powder from 12 d old seedlings was mixed with a grinding buffer similar to (80), using HEPES instead of Tricine for crosslinker compatibility. Briefly, 5 g of tissue powder was ground in a mortar and pestle with 30ml of grinding buffer [20mM HEPES-KOH pH 8.0, 330mM sorbitol, 5 mM EDTA, 5 mM EGTA, 5 mM MgCl_2_, and 10 mM NaHCO_3_] at 4°C, and under green illumination only. The resuspended homogenate was filtered twice through two layers of Miracloth. 30 ml of the filtrate was carefully transferred onto 10 ml of 40% Percoll [20 mM HEPES-KOH pH 8.0, 330 mM sorbitol, 40% v/v Percoll, 2.5 mM EDTA, 5 mM MgCl_2_, and 10 mM NaHCO_3_] in a 50ml centrifuge tube, and centrifuged at 2,600g for 10 min at 4°C. The bottom pellet, enriched in intact chloroplasts, was collected and resuspended in 30 ml of wash buffer [20 mM HEPES-KOH pH 8.0, 330 mM sorbitol, 2.5 mM EDTA, 2 mM MgCl2, and 10 mM NaHCO_3_], and centrifuged at 2,600g for 3 min at 4°C. Each pellet derived from 5 g of tissue was washed twice with 4.5 ml of 1x PBS supplemented with MgCl_2_ [137 mM NaCl, 2.7 mM KCl, 10 mM Na2HPO_4_, 1.8 mM KH_2_PO_4_, and 2.5 mM MgCl_2_] and then resuspended in 1mL 1x PBS supplemented with MgCl2 for the cross-linking reaction. This process was repeated across multiple batches, with a total of 195 g of tissue used for cross-linking.

#### Nuclear isolation:

The method for nuclear isolation was adapted from (81) with slight modification. Powdered dark-growth Arabidopsis tissue of approximately 2 g was completely homogenized with lysis buffer [20 mM HEPES (pH 7.4), 20 mM KCl, 2 mM EDTA, 2.5 mM MgCl_2_, 25% glycerol, 250 mM sucrose, 1 mM dithiothreitol, 1 mM phenylmethylsulfonyl fluoride (PMSF), 1× protease inhibitor cocktail, and 0.2% formaldehyde] at tissue:buffer ratio of 1:5 (g/mL, w/v). The homogenate was then filtered through a 40 μm cell strainer and centrifuged at 4°C, 1,500g for 10 min. The pellet was retained as the nuclear fraction. The nuclear pellet was then washed 5 times with 5 mL of nuclear washing buffer [20 mM HEPES (pH 7.4), 2.5 mM MgCl_2_, 25% glycerol, 0.2% Triton X-100, 1 mM PMSF, and 1× protease inhibitor cocktail] and centrifuged at 4°C, 1,500g for 10 min to collect the pellets after each wash. The washed nuclear pellet was then resuspended in 0.5 mL of nuclear resuspension buffer 1 [20 mM HEPES (pH 7.4), 10 mM MgCl_2_, 250 mM sucrose, 0.2% Triton X-100, 1 mM PMSF, and 1× protease inhibitor cocktail] and carefully laid on top of nuclear resuspension buffer 2 [20 mM HEPES (pH 7.4), 10 mM MgCl_2_, 1.7 M sucrose, 0.2% Triton X-100, 1 mM PMSF, and 1× protease inhibitor cocktail], then centrifuged at 4°C,16,000g for 45 min. The supernatant was carefully removed, and the nuclear pellet was resuspended in 0.5 mL 1 X PBS (pH 7.5) buffer for crosslinking reaction. The intactness and purity of the nuclear pellet was assessed by DAPI staining under a Leica SP8 confocal microscope. Multiple samples were prepared in parallel, and final isolated nuclei were pooled before crosslinking.

### Cross-linking reaction

PhoX or tBU-PhoX cross-linker was freshly prepared at a stock concentration of 100 mM in DMSO for each cross-linking experiment (supplementary Fig. S3). A working concentration of 2–5 mM cross-linker was added to buffer solution containing whole cell lysate, isolated chloroplasts or isolated nuclei and incubated for 2 h at 4°C (dark condition for chloroplast cross-linking) with end-to-end rotation. The cross-linking reaction was quenched by adding Tris pH 8.0 to a final concentration of 20 mM and subjected to protein extraction.

### Protein extraction, digestion, IMAC enrichment and RPLC fractionation

Protein extraction from the cross-linked samples, digestion to peptides, immobilized metal affinity chromatography (IMAC) enrichment, and high pH reverse HPLC fractionation followed the same procedure as described in (13). Multiple batches of PhoX-cross-linked peptides from isolated intact chloroplasts were combined before IMAC enrichment. More information can be found in supplementary Fig. S3. Multiple batches of samples were combined prior to high pH reversed-phase HPLC offline fractionation (RPLC) on Vanquish Flex HPLC, and fractions were collected for further LC-MS/MS analysis.

To generate selected proteomic libraries, total peptides from cell lysate and chloroplast samples were analyzed by LC-MS/MS. Chloroplast peptides were further fractionated into eight fractions using the Pierce High pH Reverse-Phase Peptide Fractionation Kit.

### diGly proteomics analysis to identify ubiquitinated proteins *in vivo*

Metabolically labeled Arabidopsis plants with ^14^N and ^15^N isotope were harvested, followed by protein extraction and trypsin digestion as previously described (13, 65). Ubiquitinated peptides were enriched using the PTMScan^®^ Ubiquitin Remnant Motif (K-ε-GG) Kit (Cell Signaling Technology) according to the manufacturer’s instructions. The enriched peptides were then analyzed by LC-MS/MS.

### LC-MS/MS analysis

Cross-linked peptides were analyzed by liquid chromatography-tandem mass spectrometry (LC-MS/MS) using an Easy-nLC 1200 ultra-performance liquid chromatography (UPLC) system (Thermo Fisher) coupled to an Orbitrap Eclipse Tribrid quadrupole Orbitrap mass spectrometer (Thermo Fisher). Peptides were first loaded onto a C18 trap column (Acclaim PepMap 100 C18) and then separated on an analytical C18 column (Aurora Series, 25 cm × 75 μm ID, Ion Opticks). The flow rate was set to 300 nL/min and the separation was performed over a 120-minute gradient. Peptides were eluted using a gradient from 3% to 28% solvent B over 106 minutes, followed by an increase from 28% to 44% over 15 minutes and a final wash at 90% solvent B for 14 minutes. Solvent A consisted of 0.1% formic acid and solvent B consisted of 80% (v/v) acetonitrile with 0.1% (v/v) formic acid.

Data was acquired on an Orbitrap Eclipse mass spectrometer. Full MS scans were acquired over a mass-to-charge (m/z) range of 375–1600 at a resolution of 120,000, with an automatic gain control (AGC) target of 200,000, a maximum injection time of 50 ms, normalized AGC target set to 50%, and RF lens set to 30%. The most intense multiply charged precursors (charge states 2–8) were selected for fragmentation with a cycle time of 3 seconds. MS/MS scans were acquired at a resolution of 15,000 with an AGC target of 5 × 10⁴, maximum injection time of 22 ms, isolation window of 1.4 m/z, and normalized AGC target set to 100%. Fragmentation was performed using higher-energy collisional dissociation (HCD) with a normalized collision energy (NCE) of 27. Dynamic exclusion was enabled for 30 seconds. For cross-linked peptide detection, charge states 3 to 8 were used.

For Real-time library search (RTLS) acquisition (15), a low-resolution MS2 survey scan performed in the ion trap over an m/z range of 120–500 was included. The resulting MS2 peaks were matched against a custom spectral library containing signature peak patterns characteristic of mono-links and cross-links (signature m/z values: 201.1231, 215.1387, 312.0632, and 377.1269, each with characteristic relative intensities for mono- or cross-linked species). Only precursor ions whose survey MS2 spectra matched the cross-link pattern using the “Similarity Search” function were selected for high-resolution MS2 acquisition.

### Data search using pLINK2 and Protein Prospector

Liner peptide searches were searched using Protein Prospector against Arabidopsis TAIR database as described in (65). Precursor and fragment mass tolerance were set up as 10 and 20 ppm.

Crosslinked peptide searches were performed using pLink 2.3.11 with the following parameters: crosslinker set to PhoX (209.972 Da); peptide length range: 6–60 amino acids; enzyme: trypsin with up to 3 missed cleavages. Fixed modification: Cys carbamidomethylation; variable modifications: protein N-terminal acetylation and Met oxidation. Precursor and fragment mass tolerances were set to 5 ppm and 20 ppm, respectively.

MS2 spectra were searched against the TAIR 10 protein database (35,386 entries), as well as selective database for cell lysate (7,835 entries) and chloroplast (9,897 entries), as described in Supplementary Fig.S3. Search results were filtered at 5% FDR at the peptide spectrum matches (PSMs) level. FDRs for monolinks, loop-links, intra- and inter-protein cross-links were calculated separately using pLINK2. To compare two acquisition methods (XL-Standard [Standard] and Real-time library search [RTLS]), data were acquired on the whole cell lysate XL samples, search against TAIR10 database, and directly compared.

### Data compilation and reporting

Intra- and inter-protein cross-links were compiled from multiple experiments (Supplementary Fig. S3) and reported separately (Supplementary Table S1). The total number of cross-linked peptide pairs was determined by counting unique lysine–lysine (K–K) residue pairs along with their corresponding backbone sequences. Peptides with identical backbone sequences and the same K–K pair were collapsed into a single count, regardless of charge state or peptide backbone modifications (e.g., oxidation).

### Subcellular localization analysis of proteins using SUBA5

The subcellular localization of cross-linked proteins was analyzed using SUBA5 (https://suba.live/). Accession IDs of proteins identified in both intra- and inter-protein cross-links were submitted as input. Only the localization consensus data were used for classification. Proteins assigned to multiple compartments were counted once in each corresponding localization category when calculating total counts.

### PANTHER protein class analysis and mapping intralinks to PDB and AlphaFold structures using ComMap

Arabidopsis gene identifiers (ATG accession numbers) were used for protein class analysis using PANTHER^™^ Protein Class tool (24), which contains 210 classification terms (version 19.0, released 2024–06-20).

To map the results of intralinks against experimentally determined structures from the Protein Data Bank (PDB) and predicted structures from AlphaFold protein structure database, the protein accession IDs were first converted to UniProt IDs. The intralink information was then mapped to available structures using ComMap, a tool that enables large-scale structure-based mapping by integrating XL-MS data with existing structural models and performing distance calculations (16).

### Crosslinking data visualization

#### Cytoscape visualization:

The entire cross-link-based protein-protein interaction (PPI) network was visualized in Cytoscape (version 3.10, https://cytoscape.org/) using inter-protein cross-link data, including heteromeric and homomultimeric cross-links. Nodes were colored according to protein class information from the PANTHER classification system, and edges were colored according to the STRING interaction score (https://string-db.org/).

#### xiVIEW:

PPIs, including both inter- and intra-protein cross-links, were visualized using xiVIEW (https://xiview.org/) (82). PPIs were categorized via the built-in STRING data loader (score cutoff 0.4). Cross-linked peptides supporting the same lysine pair were consolidated into a unique K-K linkage, collapsing redundancy (e.g. due to miscleavage events) and reporting each linkage only once.

#### Mapping of crosslinks to high-resolution structures of proteins/complexes.

Intra- and inter-crosslinks were superimposed on PDB or AlphaFold structures using the built-in function of xiVIEW, which also calculated the corresponding crosslink distances within the high-resolution structures. Alignments of protein orthologs were performed and visualized using PyMOL (https://www.pymol.org/).

### Co-localization assay

Co-localization was assessed in *Nicotiana Benthamian* leaves using transient co-expression. WSD4 was cloned into a modified pMDC43 Gateway binary vector containing an N-terminal mCherry tag to generate 35S::mCherry-WSD4, and HTB11 was introduced to pGWB6 gateway vector to generate 35S::sGFP-HTB11. Agrobacterium-mediated infiltration was performed, and fluorescence signals were visualized three days post-infiltration using a Leica SP8 confocal microscope.

## Supplementary Material

Supplement 1 Table S1: Summary of cross-linked peptide, including intra- and inter- protein cross-links, PPI annotations based on STRING and mapping of intra-cross-links to known structures from PDB and predicted structure from AlphaFold.

Supplement 2 Table S2: Summary of cross-linked protein information and SUBA annotation

Supplement 3 Table S3: Analysis of protein classification using PANTHER.

Supplement 4 Table S4: Detected cross-links for ribosomal and histones PPIs, and a list of ribosome-related ubiquitinated peptides.

Supplement 5 Table S5: Names, accession numbers and description used in the figures and a glossary.

Supplement 6

## Data Availability

The mass spectrometry proteomics data have been deposited to the ProteomeXchange Consortium via the PRIDE partner repository with the dataset accession numbers PXD066234 and PXD066291. All other data is available from the corresponding author on reasonable request.

## Significance Statement

Understanding how proteins interact within living cells is essential to deciphering cellular architecture and function. However, capturing native protein-protein interactions (PPIs) on a global scale has proven technically challenging. Here, we present a proteome-wide cross-linking mass spectrometry (XL-MS) platform that can systematically map direct PPIs in plant cells without requiring transgenic manipulation. This approach reveals thousands of interactions spanning major subcellular compartments and resolves the architecture of critical protein assemblies, such as photosystems, ribosomes, and chromatin-associated connectivity. We mapped both known and novel interactions. This comprehensive dataset advances protein annotation and provides a valuable resource for investigating the dynamic organization of the plant proteome.
