# Supplementary material for: Mapping the Architecture of Protein Complexes in Arabidopsis Using Cross-Linking Mass Spectrometry
Source: bioRxiv. 2025 Jul 21:2025.04.28.651104. Preprint. [Version 2] doi: 10.1101/2025.04.28.651104 (PMC12330729; doi:10.1101/2025.04.28.651104)
Supplement: Supplement 6 [file NIHPP2025.04.28.651104v2-supplement-6.pdf]

bioRxiv preprint doi: https://doi.org/10.1101/2025.04.28.651104; this version posted July 21, 2025. The copyright holder for this preprint (which was not certified by peer review) is the author/funder, who has granted bioRxiv a license to display the preprint in perpetuity. It is made available under aCC-BY 4.0 International license.

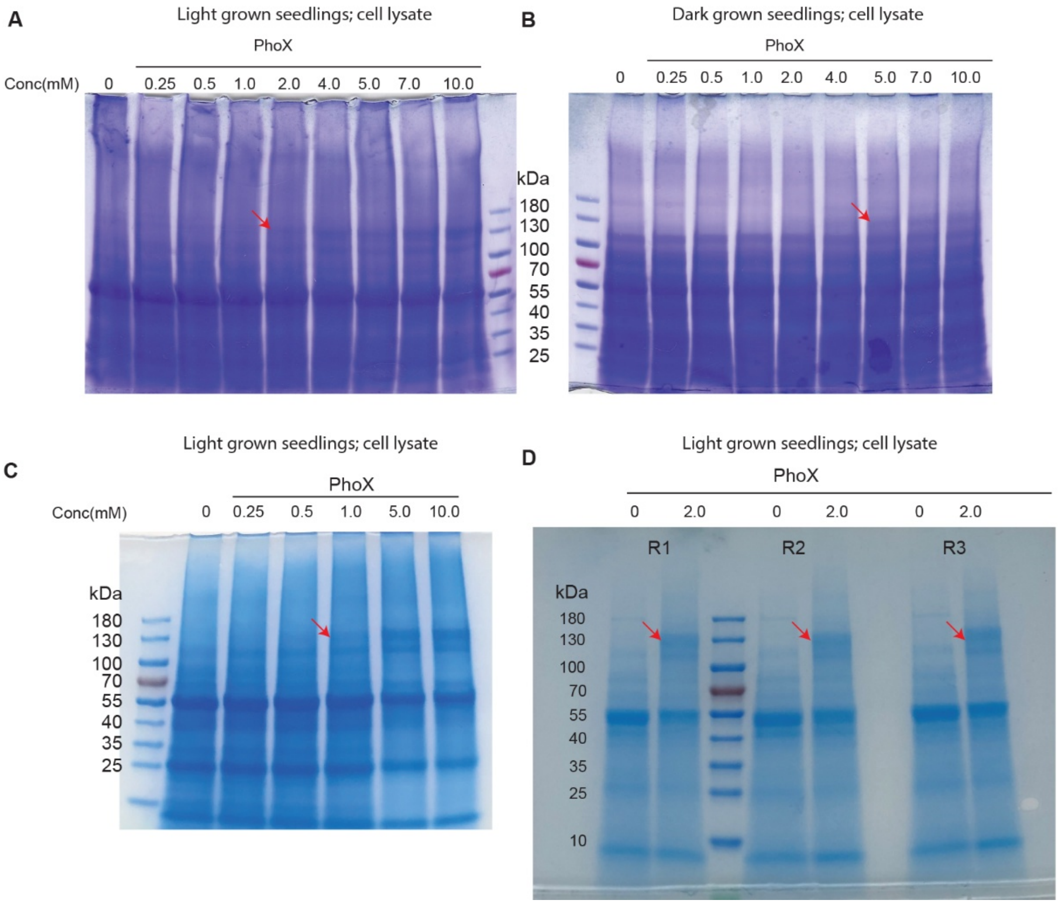

Supplementary Figure S1: Optimization of the PhoX cross-linker concentration for XL-MS.

(**A**-**D**) Different concentrations of PhoX cross-linker (0 to 10 mM) were tested on cell lysates from light (A, C, D) or dark grown seedlings (B). Increased protein cross-linking, indicated by the accumulation of higher molecular weight proteins (red arrow), was observed with increasing cross-linker concentration. However, no additional visible cross-linking was detected at higher concentrations (7-10 mM).

bioRxiv preprint doi: https://doi.org/10.1101/2025.04.28.651104; this version posted July 21, 2025. The copyright holder for this preprint (which was not certified by peer review) is the author/funder, who has granted bioRxiv a license to display the preprint in perpetuity. It is made available under aCC-BY 4.0 International license.

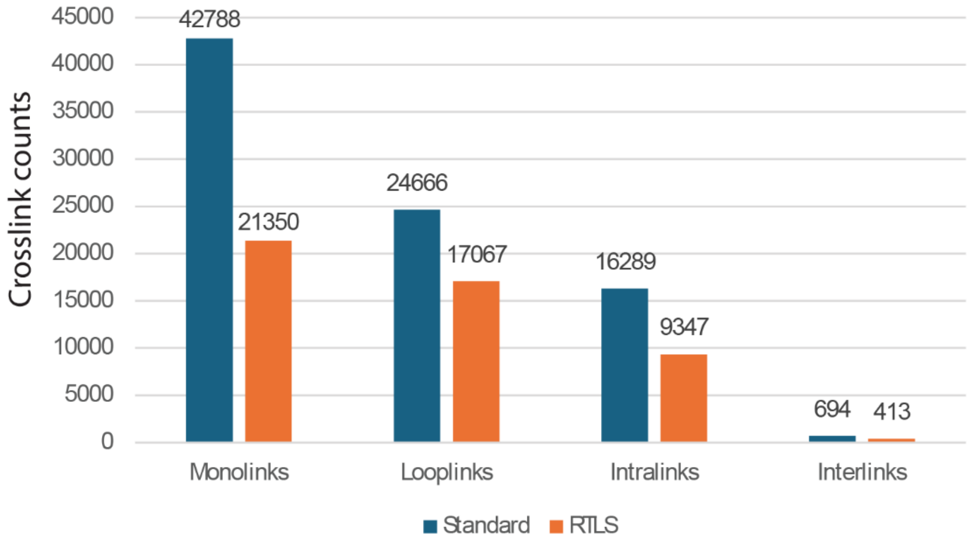

Supplementary Figure S2: **Standard XL acquisition outperforms Real-Time Library Search (RTLS) acquisition.**

Data were acquired on an Orbitrap Eclipse mass spectrometer using PhoX-enriched cross-linked peptides from Arabidopsis cell lysate samples, then searched against the entire Arabidopsis protein database. RTLS acquisition significantly reduced the identification of monolinks, where one NHS ester group is cross-linked to a peptide while the other is hydrolyzed, by approximately 50%, and loop links, which are cross-links within a single peptide, by about 31%. However, RTLS also substantially decreased the detection of inter-peptide cross-links, including both intraprotein (intralinks) and interprotein (interlinks) cross-links, by 43% and 40%, respectively.

bioRxiv preprint doi: https://doi.org/10.1101/2025.04.28.651104; this version posted July 21, 2025. The copyright holder for this preprint (which was not certified by peer review) is the author/funder, who has granted bioRxiv a license to display the preprint in perpetuity. It is made available under aCC-BY 4.0 International license.

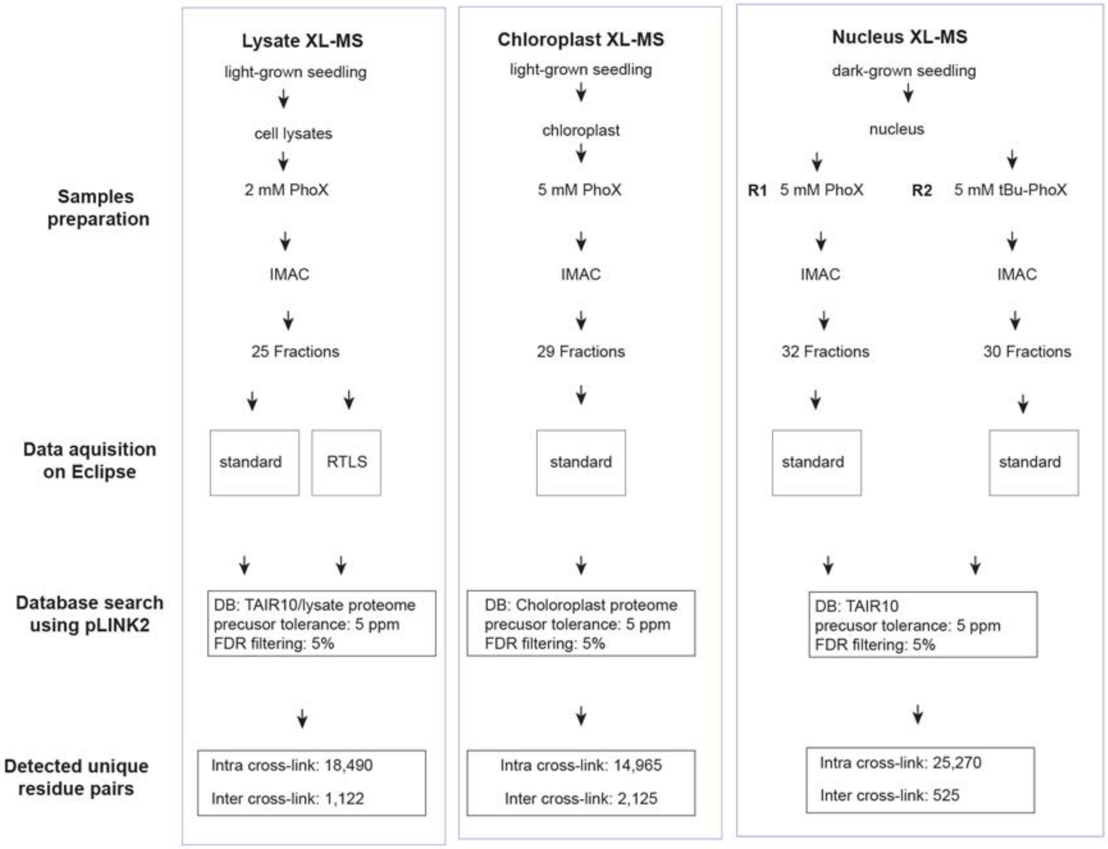

Supplementary Fig. S3: Summary of XL-MS experiments.
Key details of sample preparation, data acquisition using the Orbitrap Eclipse mass spectrometer, and database searching using pLINK2 are provided. A summary of the unique cross-linked peptide residue pairs identified across experiments is also included.

bioRxiv preprint doi: https://doi.org/10.1101/2025.04.28.651104; this version posted July 21, 2025. The copyright holder for this preprint (which was not certified by peer review) is the author/funder, who has granted bioRxiv a license to display the preprint in perpetuity. It is made available under aCC-BY 4.0 International license.

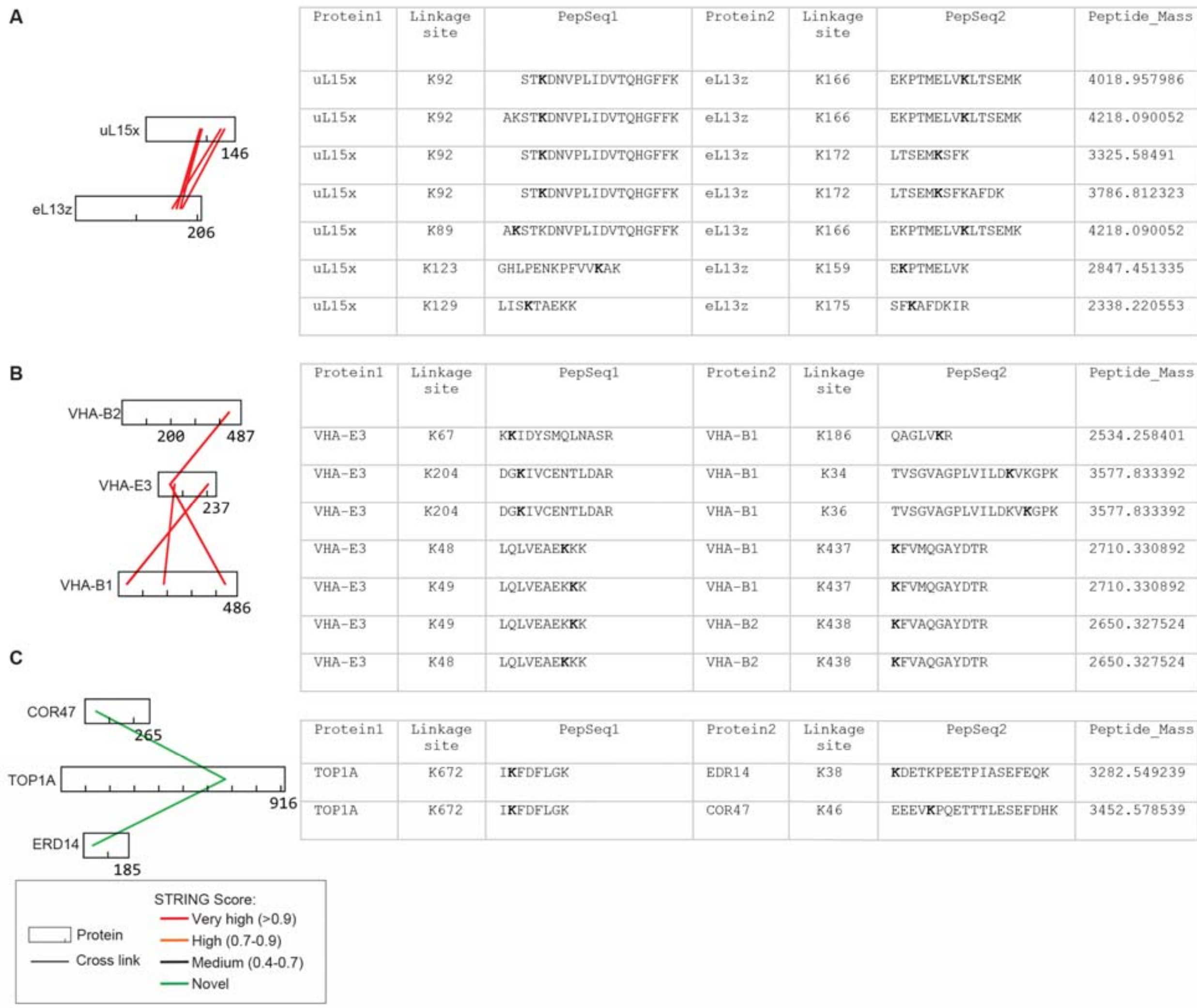

Protein1	Linkage site	PepSeq1	Protein2	Linkage site	PepSeq2	Peptide_Mass
uL15x	K92	ST**K**DNVPLIDVTQHGFFK	eL13z	K166	EKPTMELV**K**LTSEMK	4018.957986
uL15x	K92	AKST**K**DNVPLIDVTQHGFFK	eL13z	K166	EKPTMELV**K**LTSEMK	4218.090052
uL15x	K92	ST**K**DNVPLIDVTQHGFFK	eL13z	K172	LTSEM**K**SFK	3325.58491
uL15x	K92	ST**K**DNVPLIDVTQHGFFK	eL13z	K172	LTSEM**K**SFKAFDK	3786.812323
uL15x	K89	A**K**STKDNVPLIDVTQHGFFK	eL13z	K166	EKPTMELV**K**LTSEMK	4218.090052
uL15x	K123	GHLPENKPFVV**K**AK	eL13z	K159	E**K**PTMELVK	2847.451335
uL15x	K129	LIS**K**TAEKK	eL13z	K175	SF**K**AFDKIR	2338.220553

Protein1	Linkage site	PepSeq1	Protein2	Linkage site	PepSeq2	Peptide_Mass
VHA-E3	K67	K**K**IDYSMQLNASR	VHA-B1	K186	QAGLV**K**R	2534.258401
VHA-E3	K204	DG**K**IVCENTLDAR	VHA-B1	K34	TVSGVAGPLVILD**K**VKGPK	3577.833392
VHA-E3	K204	DG**K**IVCENTLDAR	VHA-B1	K36	TVSGVAGPLVILDKV**K**GPK	3577.833392
VHA-E3	K48	LQLVEAE**K**KK	VHA-B1	K437	**K**FVMQGAYDTR	2710.330892
VHA-E3	K49	LQLVEAEK**K**K	VHA-B1	K437	**K**FVMQGAYDTR	2710.330892
VHA-E3	K49	LQLVEAEK**K**K	VHA-B2	K438	**K**FVAQGAYDTR	2650.327524
VHA-E3	K48	LQLVEAE**K**KK	VHA-B2	K438	**K**FVAQGAYDTR	2650.327524

Protein1	Linkage site	PepSeq1	Protein2	Linkage site	PepSeq2	Peptide_Mass
TOP1A	K672	I**K**FDFLGK	EDR14	K38	**K**DETKPEETPIASEFEQK	3282.549239
TOP1A	K672	I**K**FDFLGK	COR47	K46	EEEV**K**PQETTTLESEFDHK	3452.578539

Supplementary Fig. S4. Representative cross-links supporting the high confidence of XL-MS analysis.

(**A**) Multiple cross-links were identified between the ribosomal proteins uL15x and eL13z, including peptide pairs with different degrees of miscleavage cross-linked at identical sites (e.g., uL15x-K92 to eL13z-K166/K172).

(**B**) Cross-links map conserved regions, with the ATPase subunit VHA-E3 (K49) cross-linked to both VHA-B1 (K437) and VHA-B2 (K438).

(**C**) Similar cross-linking patterns were observed between homologous interactions, as shown by DNA topoisomerase 1 alpha (TOP1A-K672) cross-linked to two dehydrin proteins, ERD14-K38 and COR47-K46.

bioRxiv preprint doi: https://doi.org/10.1101/2025.04.28.651104; this version posted July 21, 2025. The copyright holder for this preprint (which was not certified by peer review) is the author/funder, who has granted bioRxiv a license to display the preprint in perpetuity. It is made available under aCC-BY 4.0 International license.

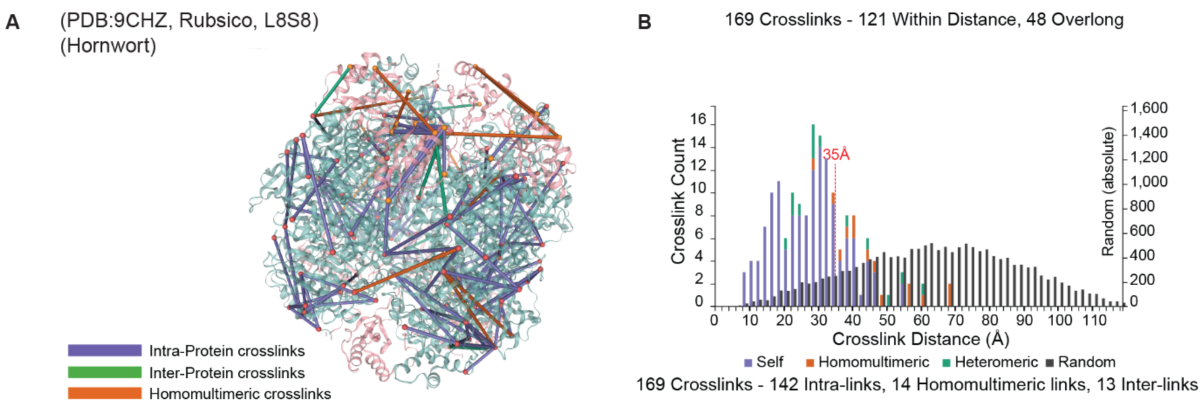

Supplementary Figure S5. Mapping of Rubisco cross-links on a high-resolution orthogonal structure of hornwort Rubisco.

Cross-links are color-coded by type: purple for self (intra)links, green for heteromeric (inter)links, and orange for homomultimeric links.

(**A**) Cross-links were mapped onto the high-resolution structure of L8S8 heterotetrameric Rubisco from hornwort (PDB: 9CHZ). RBCL subunits are shown in cyan and RBCS subunits in pink.

(**B**) Distance distribution of cross-links mapped onto Rubisco 9CHZ, showing that 71.6% of the identified cross-links fall within the expected spatial constraint of 35 Å.

bioRxiv preprint doi: https://doi.org/10.1101/2025.04.28.651104; this version posted July 21, 2025. The copyright holder for this preprint (which was not certified by peer review) is the author/funder, who has granted bioRxiv a license to display the preprint in perpetuity. It is made available under aCC-BY 4.0 International license.

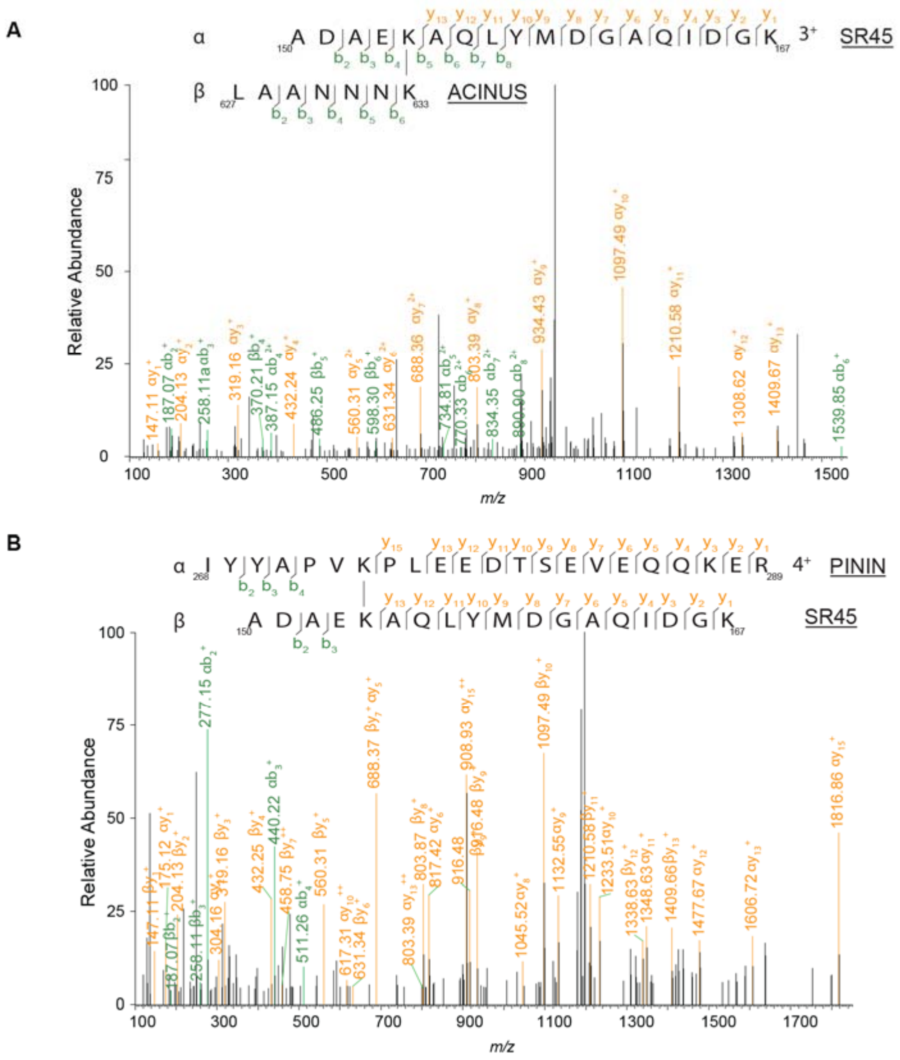

Supplementary Figure S6. High-confidence identifications of cross-linked peptides between ACINUS–SR45 and PININ–SR45.

b-ions are shown in green and y-ions in yellow. A series of fragment ions, including those from α- and β-peptides, as well as cross-linker-containing ions, were detected, supporting the reliability of the cross-link identifications.

(**A**) Spectrum of the cross-linked peptide between ACINUS-K633 and SR45-K154.

(**B**) Spectrum of the cross-linked peptide between PININ-K274 and SR45-K154.

bioRxiv preprint doi: https://doi.org/10.1101/2025.04.28.651104; this version posted July 21, 2025. The copyright holder for this preprint (which was not certified by peer review) is the author/funder, who has granted bioRxiv a license to display the preprint in perpetuity. It is made available under aCC-BY 4.0 International license.

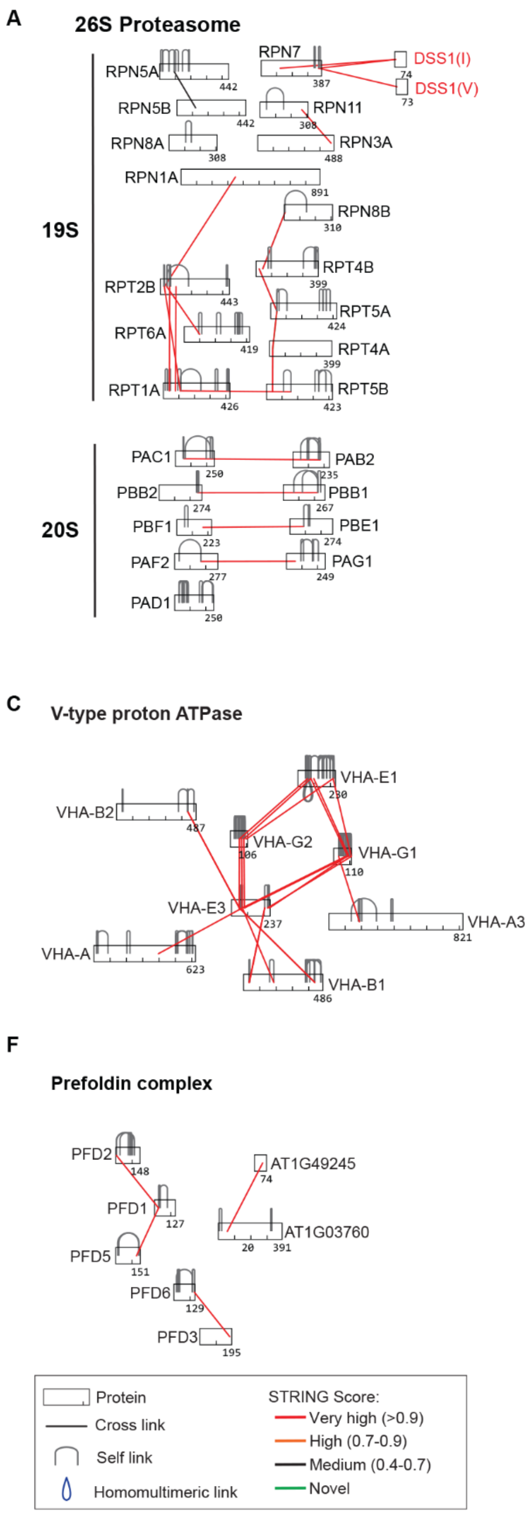

Supplementary Fig. S7. Topological mapping of multiple protein complexes by XL-MS. Proteins are shown as rectangles scaled to their respective lengths. Cross-links are color-coded based on STRING score confidence thresholds, highlighting both known and novel interactions.

bioRxiv preprint doi: https://doi.org/10.1101/2025.04.28.651104; this version posted July 21, 2025. The copyright holder for this preprint (which was not certified by peer review) is the author/funder, who has granted bioRxiv a license to display the preprint in perpetuity. It is made available under aCC-BY 4.0 International license.

(**A**) Mapping of 26S proteasome subunits, highlighting interaction interfaces between RPN7 and DSS1(I) and DSS1(V). DSS1 proteins are particularly challenging to map in cryo-EM due to their small size, intrinsic disorder, and dynamic interaction properties.
(**B**) Mapping of photosystem I (PSI) showing connectivity between different subunits.
(**C**-**E**) Mapping of different ATPase complexes, including chloroplast, V-type, and mitochondrial ATPases.
(**F**) Mapping of the prefoldin complex.
(**G**) Mapping of the phosphoenolpyruvate carboxylase (PEPC) complex.

bioRxiv preprint doi: https://doi.org/10.1101/2025.04.28.651104; this version posted July 21, 2025. The copyright holder for this preprint (which was not certified by peer review) is the author/funder, who has granted bioRxiv a license to display the preprint in perpetuity. It is made available under aCC-BY 4.0 International license.

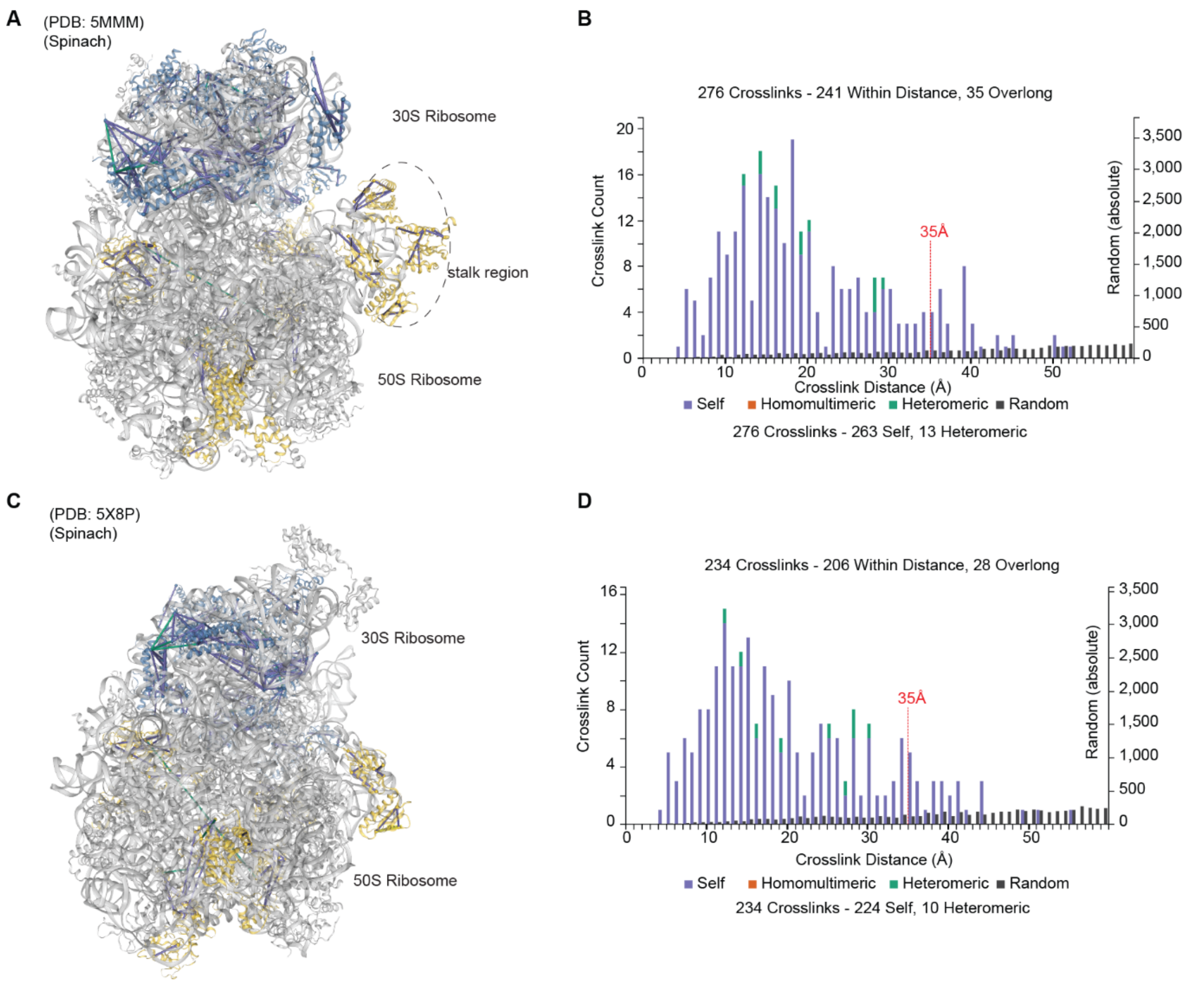

Supplementary Figure S8. Mapping of cross-links in the 70S ribosome complex onto high-resolution structures of spinach 70S ribosomes (PDB: 5MMM and 5X8P).
Cross-links are color-coded by type: purple for intralinks, green for heteromeric inter-protein cross-links, and orange for homomultimeric inter-protein cross-links.
(**A**) Structural mapping of 70S ribosome cross-links on the spinach cryo-EM structure (PDB: 5MMM), which lacks bL12 and other flexible proteins in the stalk region.
(**B**) Distance distribution of the cross-links mapped in (A). Of the 424 total cross-links detected, 276 were mapped (263 self-links and 13 heteromeric), with 241 falling within the 35 Å spatial constraint.
(**C**) Structural mapping of 70S ribosome cross-links onto an alternative spinach cryo-EM structure (PDB: 5X8P), which also lacks most of the flexible proteins in the stalk region.
(**D**) Distance distribution of the cross-links mapped in (C). Of the 424 cross-links detected, 234 were mapped (224 self-links and 10 heteromeric), with 206 falling within the 35 Å constraint.

bioRxiv preprint doi: https://doi.org/10.1101/2025.04.28.651104; this version posted July 21, 2025. The copyright holder for this preprint (which was not certified by peer review) is the author/funder, who has granted bioRxiv a license to display the preprint in perpetuity. It is made available under aCC-BY 4.0 International license.

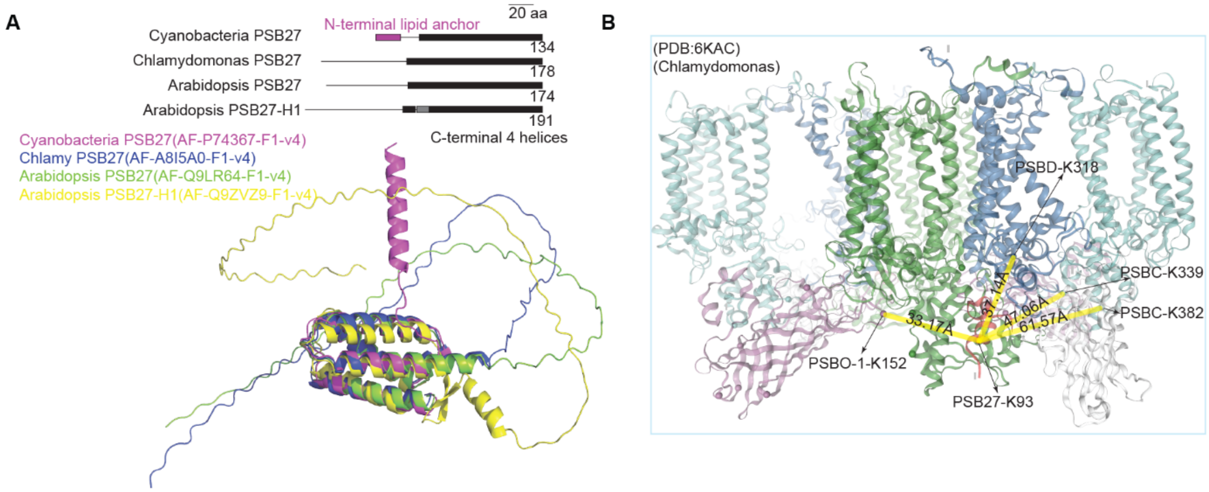

Supplementary Figure S9. Mapping of PSB27 cross-links onto a high-resolution structure of 6KAC from Chlamydomonas.

(**A**) Superimposed AlphaFold structures of PSB27 homologs reveal unique features of Arabidopsis PSB27. While it contains a conserved C-terminal four-helix bundle, Arabidopsis PSB27 lacks a lipid anchor and shows divergence in sequence and surface charge.

(**B**) Cross-links between Arabidopsis PSB27 and PSII core/oxygen-evolving complex (OEC) subunits mapped onto the Chlamydomonas PSII structure (PDB: 6KAC), which captures residues 91-114 of PSB27. The distance constraints suggest a more dynamic interaction between PSB27 and the PSII complex.

bioRxiv preprint doi: https://doi.org/10.1101/2025.04.28.651104; this version posted July 21, 2025. The copyright holder for this preprint (which was not certified by peer review) is the author/funder, who has granted bioRxiv a license to display the preprint in perpetuity. It is made available under aCC-BY 4.0 International license.

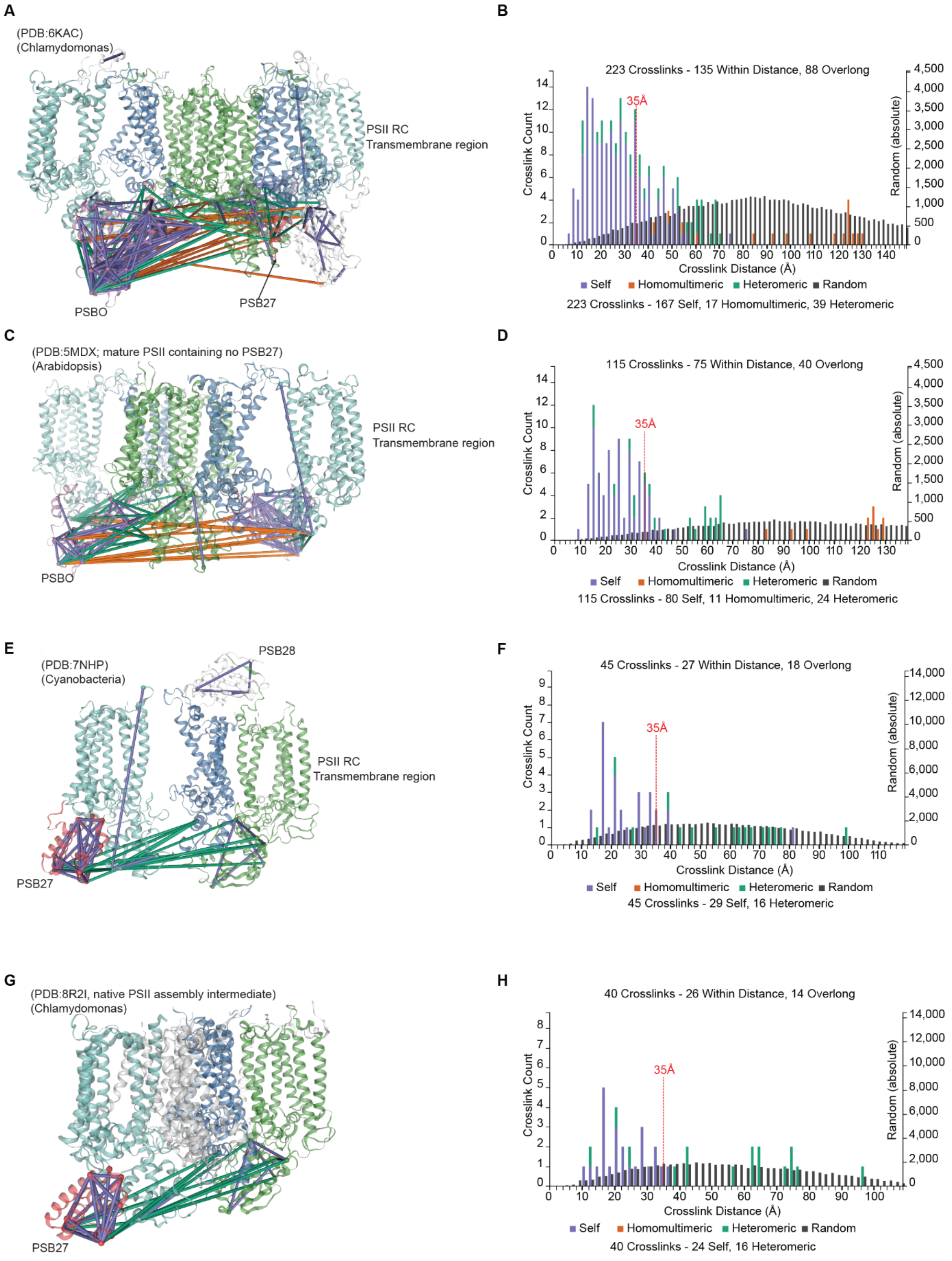

Supplementary Figure S10. Mapping of PSII complex cross-links onto several available high-resolution structures. A total of 581 cross-links were mapped, including 504 self-links

bioRxiv preprint doi: https://doi.org/10.1101/2025.04.28.651104; this version posted July 21, 2025. The copyright holder for this preprint (which was not certified by peer review) is the author/funder, who has granted bioRxiv a license to display the preprint in perpetuity. It is made available under aCC-BY 4.0 International license.

and 77 heteromeric links. Cross-links are colored according to the type of cross-link (purple for self (intra) link; green for heteromeric (inter) link; orange for homomultimeric link). The distribution of cross-link distances from the integrated cross-link model mapping is shown alongside the structural mapping.
(**A-B**) Structure mapping of PSII cross-links onto the Chlamydomonas PSII-LHCII supercomplex (PDB: 6KAC), which includes the PSII reaction center, PsbO, and a short sequence of Psb27.
(**C-D**) Structure mapping of PSII cross-links onto the Arabidopsis PSII-LHCII supercomplex, (PDB: 5MDX) which represents mature PSII, containing the PSII reaction center and PsbO but lacking Psb27.
(**E-F)** Cross-links mapped onto the cyanobacterial PSII assembly intermediate structure (PDB: 7NHP), which includes the PSII reaction center together with Psb27 and Psb28. Cyanobacteria have a single copy of the PSB27 gene. Fewer cross-links were mapped onto this assembly intermediate.
(**G-H**) Cross-links mapped onto the *Chlamydomonas* PSII assembly intermediate structure (PDB: 8R2I), representing a native assembly intermediate containing the PSII reaction center and Psb27. Similarly, fewer cross-links were mapped to this structure.

bioRxiv preprint doi: https://doi.org/10.1101/2025.04.28.651104; this version posted July 21, 2025. The copyright holder for this preprint (which was not certified by peer review) is the author/funder, who has granted bioRxiv a license to display the preprint in perpetuity. It is made available under aCC-BY 4.0 International license.

A

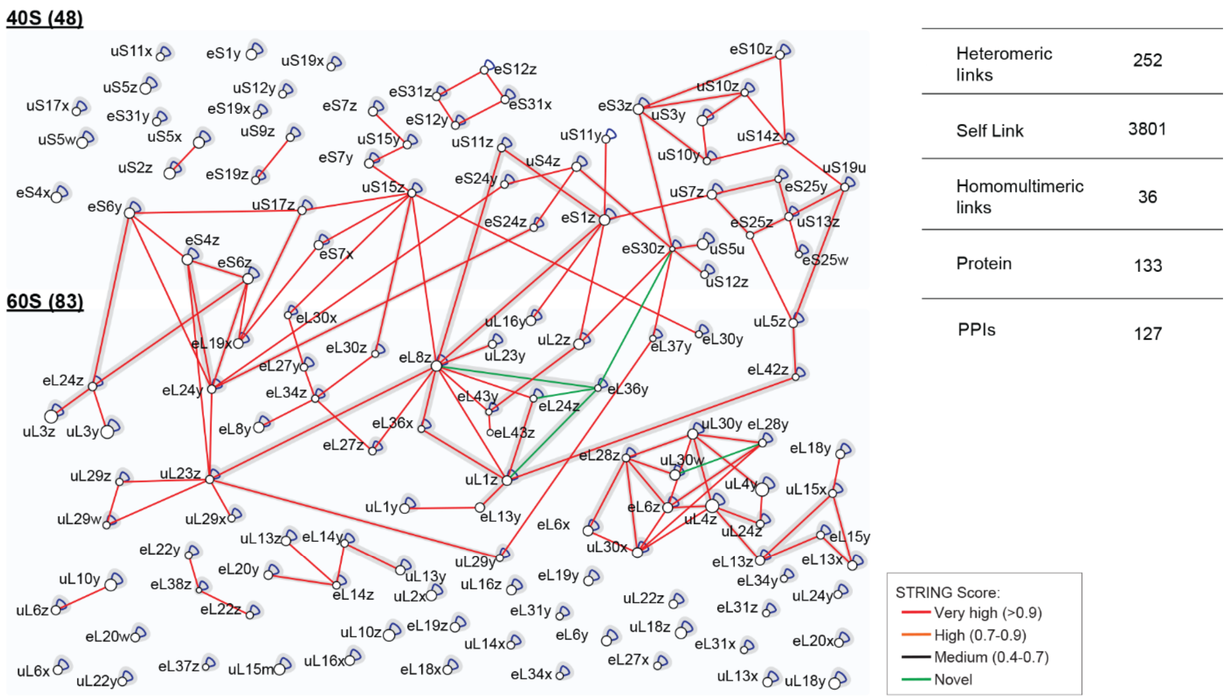

B

Heteromeric links	252
Self Link	3801
Homomultimeric links	36
Protein	133
PPIs	127

C

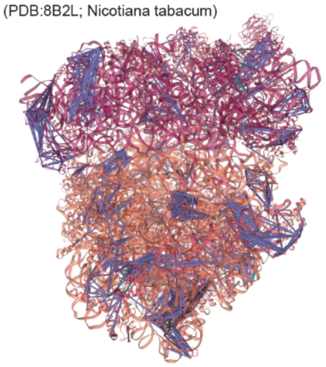

D

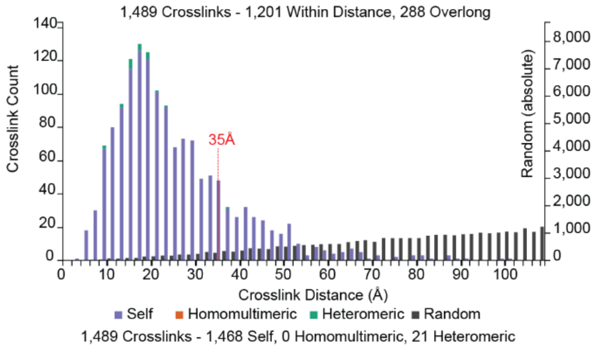

Supplementary Figure S11. Topology of the 80S ribosomal complex.
(**A**) 2D topological arrangement of the 40S and 60S ribosomal subunits based on their connectivity. Links between proteins are colored according to the confidence thresholds of the STRING score.
(**B**) Summary of cross-links and protein-protein interaction (PPI) information from (A).
(**C**) 80S ribosome cross-links mapped onto the high-resolution structure of the tobacco 80S ribosome (PDB: 8B2L). The large ribosomal subunit is colored orange and the small ribosomal subunit is colored red. Links are color-coded according to link type: purple for self-links (intralinks), green for heteromeric links (interlinks), and orange for homomultimeric links.
(**D**) Distribution of cross-link distances from the integrated cross-link model in (C).

bioRxiv preprint doi: https://doi.org/10.1101/2025.04.28.651104; this version posted July 21, 2025. The copyright holder for this preprint (which was not certified by peer review) is the author/funder, who has granted bioRxiv a license to display the preprint in perpetuity. It is made available under aCC-BY 4.0 International license.

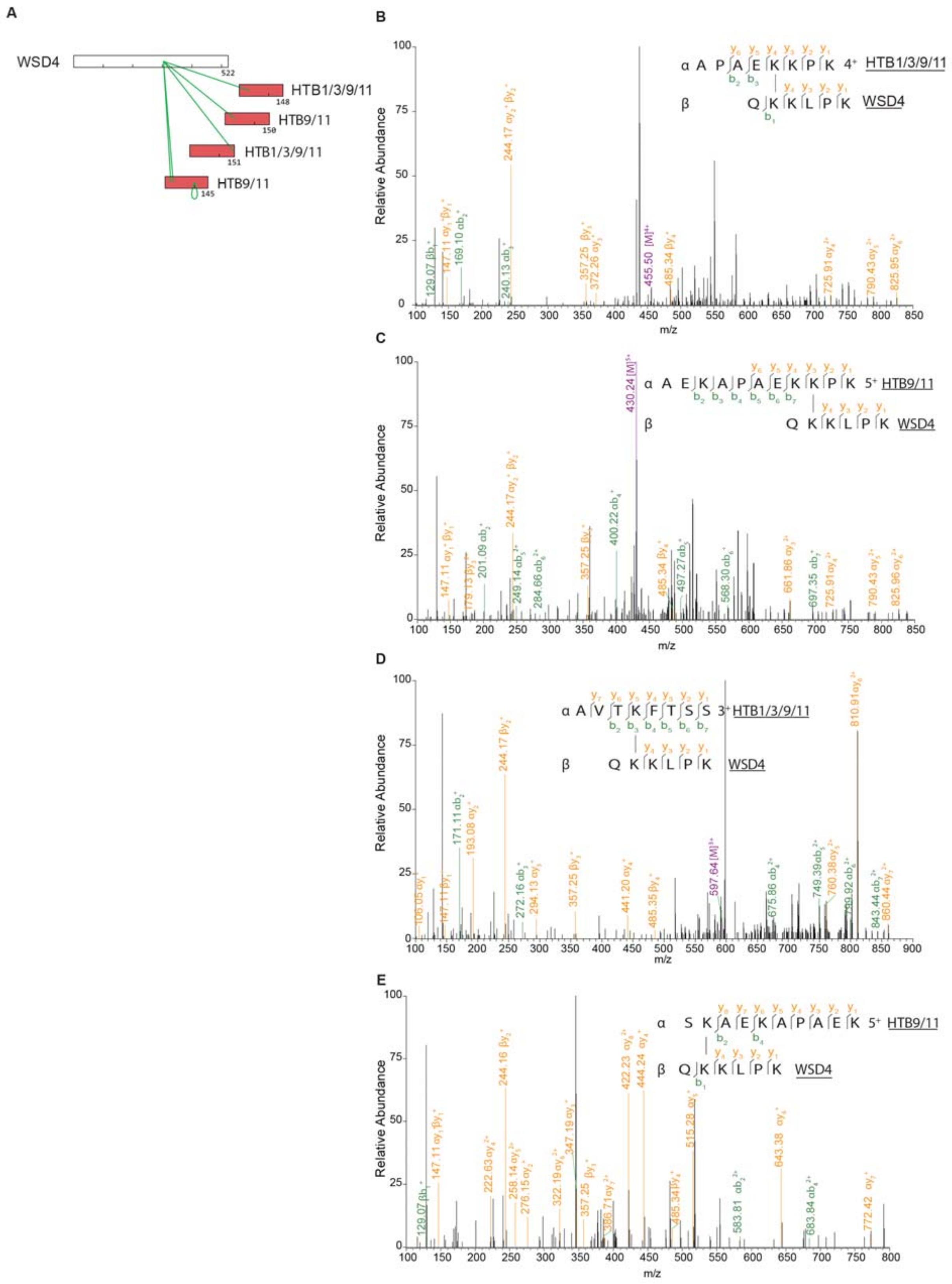

Supplementary Fig. S12. XL-MS identifies WSD4 as a novel histone interactor that associates with H2B family members.

bioRxiv preprint doi: https://doi.org/10.1101/2025.04.28.651104; this version posted July 21, 2025. The copyright holder for this preprint (which was not certified by peer review) is the author/funder, who has granted bioRxiv a license to display the preprint in perpetuity. It is made available under aCC-BY 4.0 International license.

(**A**) Cross-links were detected between WSD4 (an O-acyltransferase) and several H2B family members, including HTB1, HTB9, HTB3, and HTB11.
(**B**-**E**) High-confidence cross-link spectra reveal interactions between WSD4 and H2B family members. Series of fragment ions-including those from α- and β-peptides, as well as cross-linker-containing ions-were detected, supporting the reliability of the cross-link identifications.
